# A Numerical Comparison of Petri Net and Ordinary Differential Equation SIR Component Models

**DOI:** 10.1109/access.2025.3645087

**Published:** 2025-12-16

**Authors:** TREVOR RECKELL, BRIGHT KWAKU MANU, BECKETT STERNER, PETAR JEVTIĆ, REGGIE DAVIDRAJUH

**Affiliations:** 1 School of Mathematical and Statistical Sciences, Arizona State University, Tempe, AZ 85287, USA; 2 School of Computing and Augmented Intelligence, Arizona State University, Tempe, AZ 85287, USA; 3 School of Life Sciences, Arizona State University, Tempe, AZ 85287, USA; 4 Faculty of Science and Technology, University of Stavanger, 4021 Stavanger, Norway

**Keywords:** Petri nets, differential equations, ODE, epidemiology, modeling, RRMSE, RMSE

## Abstract

Petri nets are an increasingly used modeling framework for the spread of disease across populations or within an individual. For example, the Susceptible-Infectious-Recovered (SIR) compartment model is foundational for population epidemiological modeling and has been implemented in several prior Petri net studies. While the SIR model is typically expressed as Ordinary Differential Equations (ODEs), with continuous time and variables, Petri nets operate as discrete event simulations with deterministic or stochastic timings. We present the first systematic study of the numerical convergence of two distinct Petri net implementations of the SIRS compartment model relative to the standard ODE. In particular, we introduce a novel deterministic implementation of the SIRS model using variable transition weights in the GPenSIM package and stochastic Petri net models using Spike. We show how rescaling and rounding procedures are critical for the numerical convergence of Petri net SIR models relative to the ODEs, and we achieve a relative root mean squared error of less than 1% compared to ODE simulations for biologically relevant parameter ranges. Our findings confirm that both stochastic and deterministic discrete time Petri nets are valid for modeling SIR-type dynamics with appropriate numerical procedures, laying the foundations for larger-scale use of Petri net models.

## I. INTRODUCTION

Petri nets (PNs) are a discrete event mathematical formalism that has become an increasingly popular modeling tool in epidemiology and other areas of the life sciences. Recent applications include models of Covid-19 spread within and across countries [1], [2], pertussis vaccination [3], social-ecological systems [4], rumor propagation in social networks [5], and the behavior of network motifs [6]. Key features of the Petri net formalism include the ability to construct modular, combinatorial models [6], [7], [8]; the ease of visualizing and interpreting Petri net models for those without a strong background in mathematics; and advances in software implementations that enable large-scale simulation including deterministic and stochastic behaviors [9], [10], [11], [12]. Furthermore, recent work has highlighted the importance of statistical sensitivity analysis and stochastic stability in epidemic models, reinforcing the need for rigorous comparison between modeling frameworks [13], [14]. Recent studies have also expanded the scope of Petri net modeling to capture the complex, multi-scale dynamics of immune responses within individuals [15], highlighting the versatility of Petri nets beyond traditional population-level compartmental models. However, some essential questions remain about how Petri net implementations of basic epidemiological models such as Susceptible-Infectious-Recovered (SIR) models relate to their standard formulations using Ordinary Differential Equations (ODEs) in epidemiology. While prior theoretical results indicate that Petri net SIR models can be made to converge asymptotically to the behavior of ODE and Stochastic Differential Equation (SDE) counterparts [16], there has been little systematic investigation of the numerical conditions under which this occurs using contemporary software tools. For example, the fitting and forecasting abilities of Petri nets compared to ODEs are not considered in [8], [17], [18], and [19]. Recent theoretical frameworks have established Petri nets as a rigorous foundation for deriving key epidemiological metrics like the basic reproduction number [20], while others have successfully applied Coloured Petri Nets to model stratified pandemics with complex spatial dynamics [2]. These advances underscore the urgency of validating the numerical fidelity of Petri nets against the ODE standards used in mainstream epidemiology. Understanding precisely when and how Petri net SIR models may diverge from ODE or SDE systems is critical for large-scale combinatorial modeling efforts [6], [7], [8], [21].

In this work, we investigate the numerical convergence of two Petri net implementations to an ODE SIRS model. The second S in SIRS indicates that individuals may become susceptible again after recovering from an infection. We present a novel implementation of SIR-type models using variable arc weights for transitions in a Petri net model with deterministic time steps using the GPenSIM toolkit. We also analyze a Continuous-Time Markov Chain (CTMC) Petri net with stochastic time steps using the Spike toolkit. We evaluate the behaviors of these Petri net models to numerical solutions of ODE model using a standard MatLab differential equation solver.

We find that both Petri net models can be made to converge to within 1% relative root mean squared error (RRMSE) of the ODE using appropriate rescaling of population size in the case of the CTMC Petri Model and a combination of population size and time step in the variable arc weight model. We also find that the choice of numerical rounding procedures has a significant impact on the RRMSE of the variable arc weight model when the infected population is close to zero. Our results, therefore, provide a valuable guide for the numerical simulation of SIR dynamics under biologically realistic parameter values.

In section II-A we introduce some basics of the Petri net formalism and introduce two different ways that Petri nets can be simulated. In section II-B and II-C we discuss specific ways in how PN simulations can be improved in relation to a known system of ODEs within the software framework that we have laid out. Lastly, in section III the PN simulation methods are compared to ODEs and results of how the PN simulation improvement methods affect the computation time are shown. In section IV we conclude how the methods we laid out set the groundwork for proper implementation of Petri nets.

## II. METHODOLOGY

Our approach is based on the numerical comparison of PN models to ODEs, which are a standard modeling framework used in fields such as epidemiology, drug pharmacokinetics, and cancer biology [22], [23], [24], and [25]. The SIR model is the most commonly used in epidemiology, and it also serves as the foundation for a large family of more complex models. Kermack and McKendrick initially derived the model in 1927 [26] by structuring the total host population into repetition three different “compartments”: S denoting the susceptible population, I denoting the infected population, and R denoting the recovered population. Equations 1–3 describe the resulting system behavior, with the parameter β representing the rate at which susceptible populations become infected and γ representing the rate at which the infected population recovers per unit of time.

To ensure reproducibility of the numerical comparisons, the specific parameter ranges and initial conditions used for the reference ODE solutions and PN simulations are detailed in Table 1.

The classical SIR model assumes that there is no birth, death, immigration, or emigration, that populations mix homogeneously, that new infections are not dependent on other factors besides βSI, and that there is an exponential waiting time for events to happen in each compartment. In practice today, the original SIR model is typically used as a basis for constructing more complex models that include disease-specific dynamics and various ways of treating or preventing the disease.

For example, the SIR model can easily be adapted to the SIRS model by adding the term δR, which represents the rate at which hosts become re-susceptible per unit of time. By adding δR to the S compartment and subtracting it from the recovered population R compartment, we get Equations 1–3. Note that when δ=0, Equations 1–3 are equivalent to an SIR model.


(1) $$\frac{dS}{dt}=\delta R-\beta SI$$

(2) $$\frac{dI}{dt}=\beta SI-\gamma I$$

(3) $$\frac{dR}{dt}=\gamma I-\delta R.$$

### A. FUNDAMENTALS OF PETRI NETS

A Petri net graph, or Petri net structure, is a weighted bipartite graph [27] defined as n-tuple (P,T,A,w,x) where,

- P is the finite set of places (one type of vertex in the graph).
- T is the finite set of transitions (the other type of vertex in the graph).
- A is the set of arcs (edges) from places to transitions and from transitions to places in the graph A⊂(P×T)∪(T×P).
- $M_{0}$ is the initial state (also known as marking), $M_{0}=\left[m_{1},m_{2},\ldots ,m_{n}\right]$, where $m_{i}$ is the number of tokens in place $p_{i}$.
- w:A→{1,2,3,…} is the weight function on the arcs.
- x is a marking of the set of places P; $x=\left[x\left(p_{1}\right),x\left(p_{2}\right),\ldots ,x\left(p_{n}\right)\right]\in N^{n}$ is the row vector associated with x.

Tokens are assigned to places, with the initial assignment being the initial marking. The number of tokens assigned to a place is an arbitrary non-negative integer but does not necessarily have an upper bound. A transition $t_{j}\in T$ in a Petri net is said to be enabled if $x\left(p_{i}\right)\geq w\left(p_{i},t_{j}\right)$ for all $p_{i}\in I\left(t_{j}\right)$, where $I\left(t_{j}\right)$ is the set of input arcs from places to $t_{j}$. This allows us to define the state transition function, $f:N^{n}\times T\to N^{n}$, such that transition $t_{j}\in T$ fires if and only if $x\left(p_{i}\right)\geq w\left(p_{i},t_{j}\right)$ for all $p_{i}\in I\left(t_{j}\right)$. If $f\left(x,t_{j}\right)$ is defined, then we set $x^{\prime }=f\left(x,t_{j}\right)$, where $x^{\prime }\left(p_{i}\right)=x\left(p_{i}\right)-w\left(p_{i},t_{j}\right)+w\left(t_{j},p_{i}\right),i=1,\ldots ,n$. In simple terms, a transition is enabled if the number of tokens in all places connected to that transition via an incoming arc is greater than or equal to the arc weight for the respective arc connected to the transition.

In order to be precise about the relative time scales of firing events in PN models versus ODE models, we define τ to be the number of steps per unit of time in a Petri net with deterministic time steps and firings. The graph then can be defined as (P,T,A,w,x,τ) as applied to the deterministic implementation of a Petri net used in GPenSIM [9], [28], where $\frac{1}{\tau }$ is called sampling frequency and Davidrajuh defines τ as the continuity of the sequence of execution.

**FIGURE 1.**
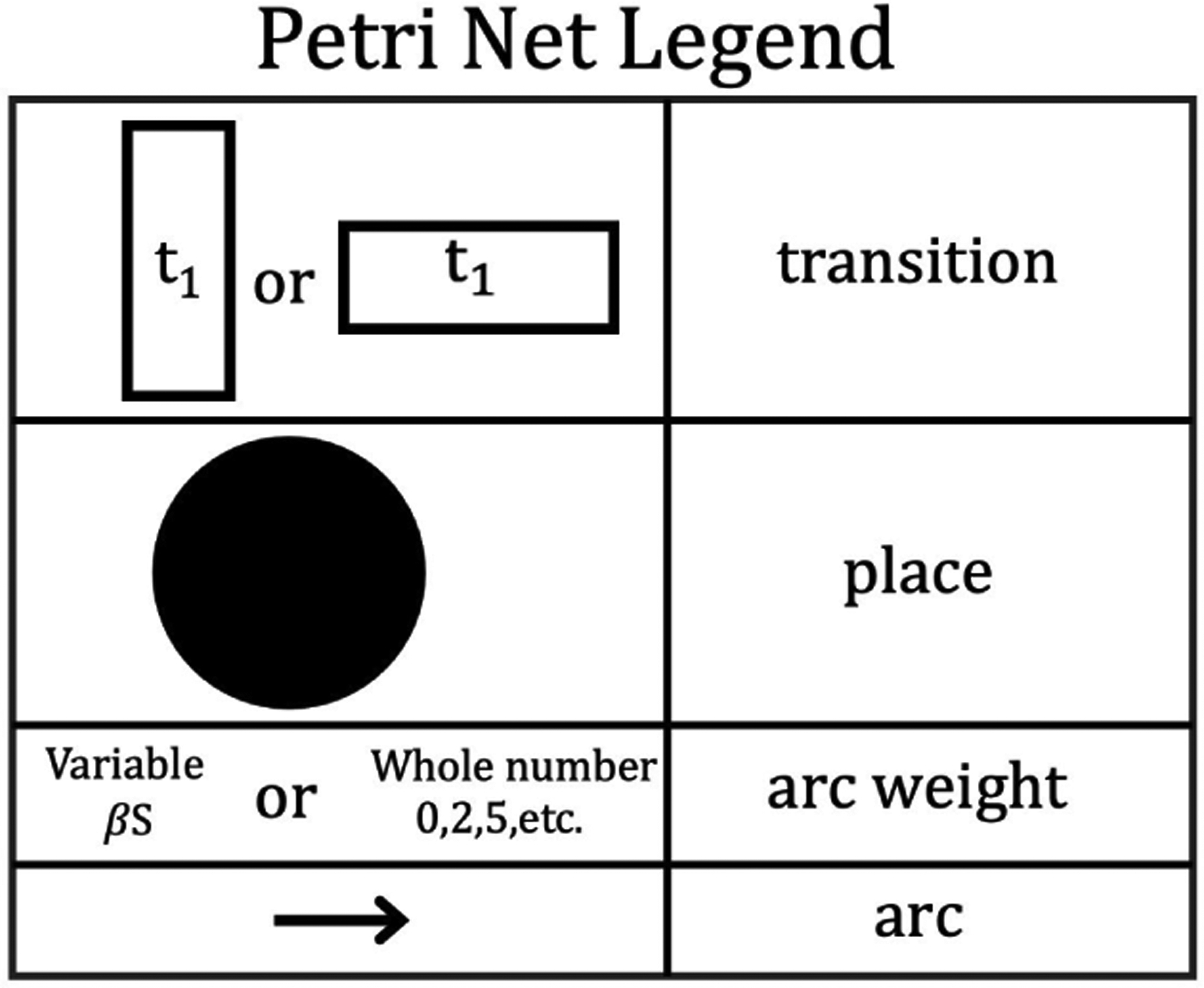
Petri nets model formalism elements.

The last fundamental element of Petri nets for our purposes is how the diagrams are drawn. We use the visual elements shown in Figure 1 to describe the logic of both deterministic Petri nets with variable arc weights and stochastically firing Petri nets with fixed arc weights.

### B. PETRI NET IMPLEMENTATIONS

A variety of Petri net simulation and software tools are freely available online, all with relative advantages and disadvantages [29], [30]. The most commonly used tools, such as GPenSIM, Snoopy, CPNTools, and Spike, can handle a range of different Petri net types. We will focus on two tools, GPenSIM and Spike, based on their robust features for scalable modeling and simulation analysis and command-line interfaces for scripting.

#### 1) GPENSIM

The General-purpose Petri net Simulator (GPenSIM) package is a new tool for the MATLAB platform. GPenSIM is designed for modeling, simulation, and performance analysis of discrete systems. There were many reasons for developing GPenSIM [9] with the primary motivation being to create a system that is easy to learn and use for both industrial and academic applications. Interoperability was also a priority so that GPenSIM can model discrete systems in different domains such as Production and Mechanical Engineering, Industrial Engineering, Computer Science and Engineering, etc., taking full advantage of MATLAB’s numerous functions in diverse toolboxes. Another goal was to provide a simple core engine with an extensible interface so that users can modify GPenSIM functions or create new functions to model their systems.

The initial version of GPenSIM was developed for simple analyses of place/transition Petri nets [9]. Later, coloring capability (Colored Petri nets) was added so that real-life systems could be modeled [11]. For modeling large real-life systems, modular modeling capability was added [10].

Petri nets implemented by GPenSIM are fundamentally deterministic, and GPenSIM allows Petri nets with non-variable arc weights, which we call static Petri nets. These static Petri nets are defined as to have arcs that are constant throughout the entire simulation. At the start of the simulation, a static Petri net graph must be defined in a separate file (Petri net Definition File (PDF)), and this file will be used throughout the simulation. The limitations of a static Petri net can be overcome using a variable arc weight Petri net implemented using an iterative approach as follows. One starts with a static Petri net graph with some initial arc weights (initial definition file). The PN simulation is run for one time step, and the resulting places markings are then used to calculate updated arc weights, which are fed into a new PDF file for the next iteration. Since the PN definition file must be recreated for each iteration step, execution time may be an important issue to consider.

#### 2) SPIKE

Spike is a Petri net simulation framework designed to model dynamic systems using stochastic and deterministic approaches. It emphasizes reproducibility for stochastic simulations by using repeatable configuration files, denoted by the “.spc” file type [12]. Spike also supports diverse simulation methods for Continuous-Time Markov Chain (CTMC) processes, including Gillespie’s direct method for exact stochastic modeling [31], tau-leaping and delta-leaping for efficient approximations [32], and deterministic ODE solvers for large-scale systems. By definition, a CTMC describes a discrete set of system states where the future state of a system depends only on its current state (memoryless), and the time between transitions to another state follows an exponential distribution.

Spike also has multiple notable features and capabilities [12]. Efficient simulation is one important feature, since Spike supports the automated execution of large sets of simulations, which can be run sequentially or in parallel. Spike also supports the simulation of stochastic, continuous, and hybrid Petri nets, accommodates colored and uncolored variants, and leverages its integration with the broader PetriNuts software framework. Spike is also designed to support various use cases, including benchmarking, adaptive model simulations, parameter scans, and model optimization. However, the underlying code of Spike is not publicly available, which limits one ability to inspect and debug the exact steps being executed by different functions.

### C. PETRI NET SIRS MODELS IN SPIKE AND GPENSIM

**FIGURE 2.**
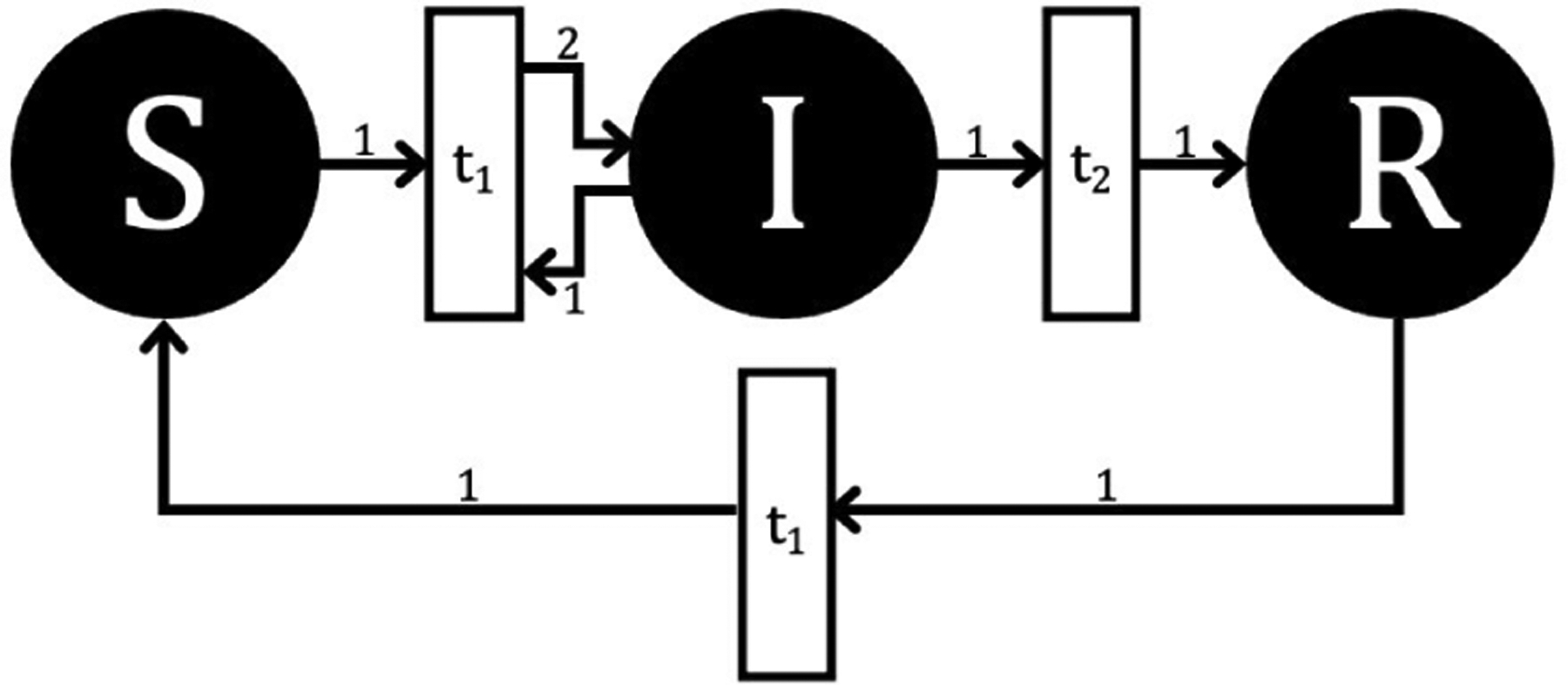
Continuous-time, Markov Chain SIRS model with stochastic firing times and fixed arc weights, as implemented in Spike.

**FIGURE 3.**
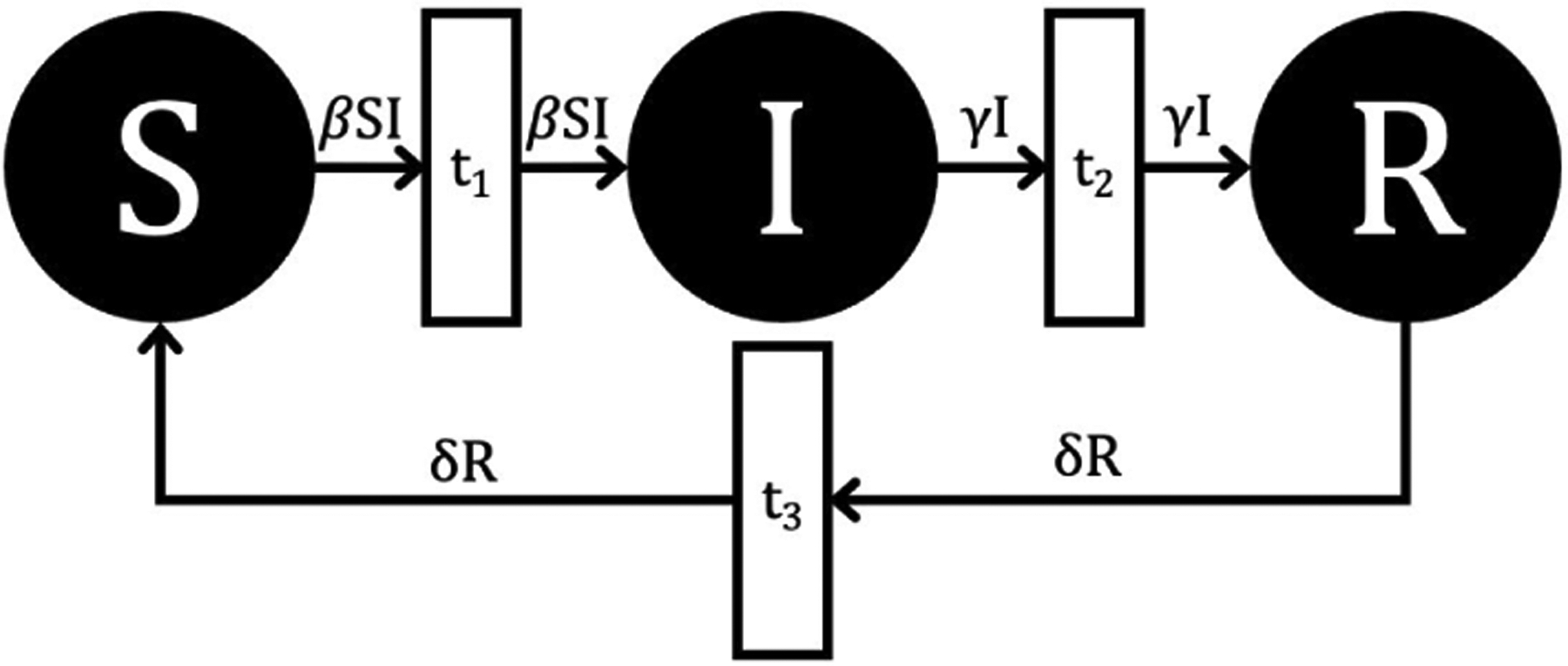
Discrete-time, deterministic SIRS model using fixed time steps and variable arc weights, as implemented in GPenSIM.

We now turn to describe the SIRS model in both a deterministic, variable arc weight form using GPenSIM and a stochastic, fixed arc weight form using Spike. In both cases, a PN model can be stated that preserves the corresponding ODE model’s assumptions (such as waiting times and closed population) and biological dynamics (such as positive populations and susceptible populations always able to be infected). However, the implemented PN models have different theoretical arcs, arc weights, and resulting dynamics. Figures 2 and 3 illustrate these differences relative to the SIRS ODE Equations 1–3.

The layout in Figure 2 reflects the application of the mass action equation from chemical reactions to determine the pattern of arcs and weights that correspond to the ODE system [33], [34]. Although the arc weights are shown as static, the frequency at which the arcs fire is dynamic as a function of βSI over time because the Petri net is implemented as a CTMC. The arc weights themselves in this layout therefore describe only the stoichiometry of tokens from each place involved in a transition.

Shifting to a deterministic PN, we have fixed time steps and the arc weights change dynamically with each time step. A key change from the CTMC layout in Figure 2 is the removal of the arc from place I to transition $t_{1}$. Additionally, the arc weights are now a function of time since their value at a time point depends on the number of tokens in one or more compartments, and they directly represent the flow of tokens between compartments instead of the stoichiometry of reactions. This shift in semantics is matched by having the deterministic PN fire on uniformly separated, discrete time points instead of stochastically in continuous time.

### D. NUMERICAL FEATURES OF DETERMINISTIC PN MODEL

We describe three numerical features that need to be addressed for the novel deterministic implementation of the SIRS model using GPenSIM: handling of discrete population sizes, rounding of non-integer arc weights, and choice of simulation time step τ.

#### 1) DISCRETE POPULATION SIZES

Since Petri net implementations in GPenSIM use discrete token values for the SIR compartments, this may disable the firing of transitions even when the arc weights and place markings are both non-zero, leading the Petri net dynamics to diverge from the corresponding ODE solution. For example, the arc going into transition 1 has a variable arc weight of βSI (see Figure 3). If S=1 and $I>\frac{1}{\beta }$, this arc will not fire even though the one susceptible individual should in principle become infected. We consider two solutions for this biologically implausible scenario.

**FIGURE 4.**
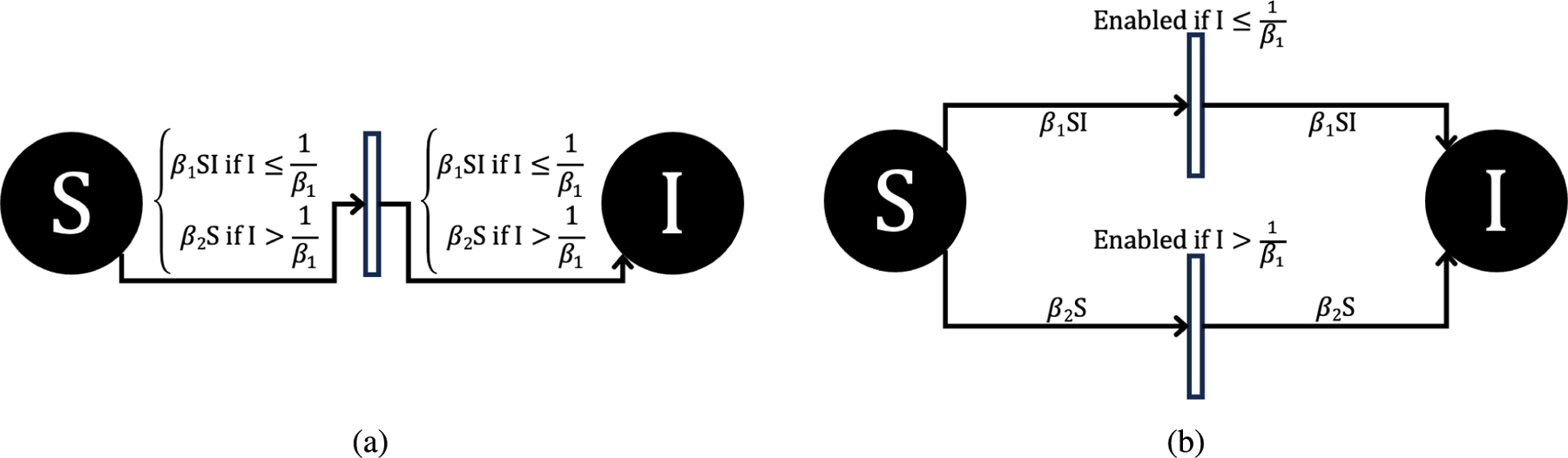
Two options for addressing arcs that become disabled in the deterministic PN model in biologically implausible ways. In (a), this can be achieved with a simple “if else” logic statement. In (b), an additional transition needs to be installed.

For example, using the options outlined in Figure 4, we can allow for the transition to fire even if the arc weight is initially higher than the place has tokens, specifically if the system is calling for a number of tokens to be moved from place S (susceptible population) to place I (infected population), which is larger than the number of tokens in place S. The transition would normally not fire until enough tokens have built up in place S (susceptible population) or the arc weight has changed to a lower value. However, this does not follow the biological dynamics described in the reference ODE model. First, we define $\beta ,\beta_{1}$, and $\beta_{2}$ to have a range of [0, 1], as they represent the proportion of individuals who become sick through contact with an infected individual. Thus, we can adapt either 4a or 4b implementations to allow a firing to occur still, since in both implementations, $\beta_{2}S$ is always greater than S, since $\beta_{2}$ is defined as less than one. In Figure 4a, we allow the formula for the arc weight to switch when the transition is disabled, where this transition being disabled corresponds biologically to not being able to infect the susceptible population. The formula goes from $\beta_{1}SI$ to $\beta_{2}S$ where $\beta_{2}=\frac{1}{I+1}$. If $\beta_{2}\leq \frac{1}{I}$, then the transition will be enabled, but the formula for $\beta_{2}$ could be set to anything in the range of $\left[0,\frac{1}{I}\right]$. The formula will depend on the system being modeled and the desired dynamics. The method laid out in Figure 4b is similar, just with a different transition being enabled if $I>\frac{1}{\beta_{1}}$, rather than the formula for the arc weight changing for the same transition. Another method of dealing with these extreme values is outlined in the PN time steps per unit time in Section II-D3. Additionally, population scaling can mitigate the effect this issue has on overall results as seen in Section III-C.

#### 2) INTEGER ARC WEIGHTS

Petri net simulation software, as a whole, does not have a standardized method for rounding dynamic arc weights, also called repetition arc expressions in some software documentation [35], [36]. Using the standard PN approach, rounding the arc weight is necessary to have the arc weights be positive integers. If positive integer arc weights is difficult or unreasonable for the Petri net application, one could use continuous weights and token values. Unfortunately, the software options for building scalable, complex models with continuous value arc weights are minimal. Among dynamic arc weight PN software with discrete arc weight values, there is no standardized rounding system for the dynamic arc weights. With this in mind, the question arises about what rounding system should be used. The initial functions we will explore are the ceiling function (weights are rounded up to the nearest positive integer), floor function (weights are rounded down to the nearest positive integer), and standard rounding (weights are rounded down to the nearest positive integer when the tenths place is less than five and up to the nearest positive integer when the tenths place is greater than or equal to five).

In addition, we propose another rounding method that utilizes standard rounding with the addition of carrying the residual of the rounding process to the next time step. In the next time step, the residual is added to the same arc’s weight before that weight is rounded again. This process is repeated each time the dynamic arc weight is recalculated. This process could be done with other rounding functions, like ceiling or floor functions, and we will compare this “standard + residual” method with many other possible rounding methods more rigorously in future work. These rounding methods will each likely have their own drawbacks in terms of computation time and memory, but our primary goal here is for the resulting PN to most closely mimic that of the reference ODE.

#### 3) TIME SCALES

When changing τ in the deterministic PN, the other parameters implemented are dependent on a respective time unit, so they need to be scaled to ensure they are applied appropriately. For instance, if the susceptible population becomes infected at a rate of 5% per unit of time, β=0.05, and we are changing the PN time step to twenty time steps per one unit of ODE time, then we need to scale β by $\frac{1}{20}$ giving $\beta =0.05\cdot \frac{1}{20}=0.0025.$ With the lower parameter value, this method also has the benefit of avoiding the situation of not allowing the transition to fire as frequently as laid out in Section II-D1.

With either method, varying time steps or firing times, there will be a significant trade off in computation time. As such, we will calculate the mean computation time when running the model for various time steps to gauge what time step level is necessary given the desired dynamic level and computational power available.

### E. COMPARISON OF PETRI NET TO ODE MODELS

We will compare the behaviors of both PN models to the ODE system under the full permitted range of the rate parameters β,γ, and δ. For simplicity, our code follows the notation for root mean square error used in MATLAB. We denote the observed data vector as A, where $A_{i}$ is a single vector entry indexed by the time point i. The forecasted data is denoted as F, where $F_{i}$ is a single forecasted vector entry indexed by the time point i. Finally, n represents the total number of time points being compared. The RRMSE formula is:

$$\text{RRMSE}=100\cdot \sqrt{\frac{\frac{1}{n}\sum_{i=1}^{n}{\left|A_{i}-F_{i}\right|}^{2}}{\sum_{i=1}^{n}{\left|A_{i}\right|}^{2}}}$$

where $A_{i}$ is the ODE model data, and $F_{i}$ is the forecasted PN model data in our application.

We aim to understand how the RRMSE is affected by rescaling key model parameters, specifically the total population size N in both models and the base time step τ in the deterministic model specifically. These parameters are known to control the relative asymptotic convergence of PN, ODE, and SDE systems for the SIR model [16]. Since the deterministic, dynamic arc weight implementation of the SIRS model is novel in the PN literature, we give more attention to the details of its implementation than the Spike CTMC model. In particular, we will discuss different rounding strategies for the variable arc weights.

## III. RESULTS

Before comparing the deterministic PN model to the reference ODE system, it is necessary to first address which rounding method is best, since this will affect how the model behaves under rescaling of total population size N and time step τ.

### A. ARC WEIGHT ROUNDING IN GPENSIM MODEL

We tested the various rounding methods for parameters β,γ, and δ, rate of infection, rate of recovery, and rate of re-susceptibility respectively, as laid out in the SIRS model for ODE and PN at extreme values, including (0,0,0) and (1,1,1), as well as intermediate, biologically plausible values.

**FIGURE 5.**
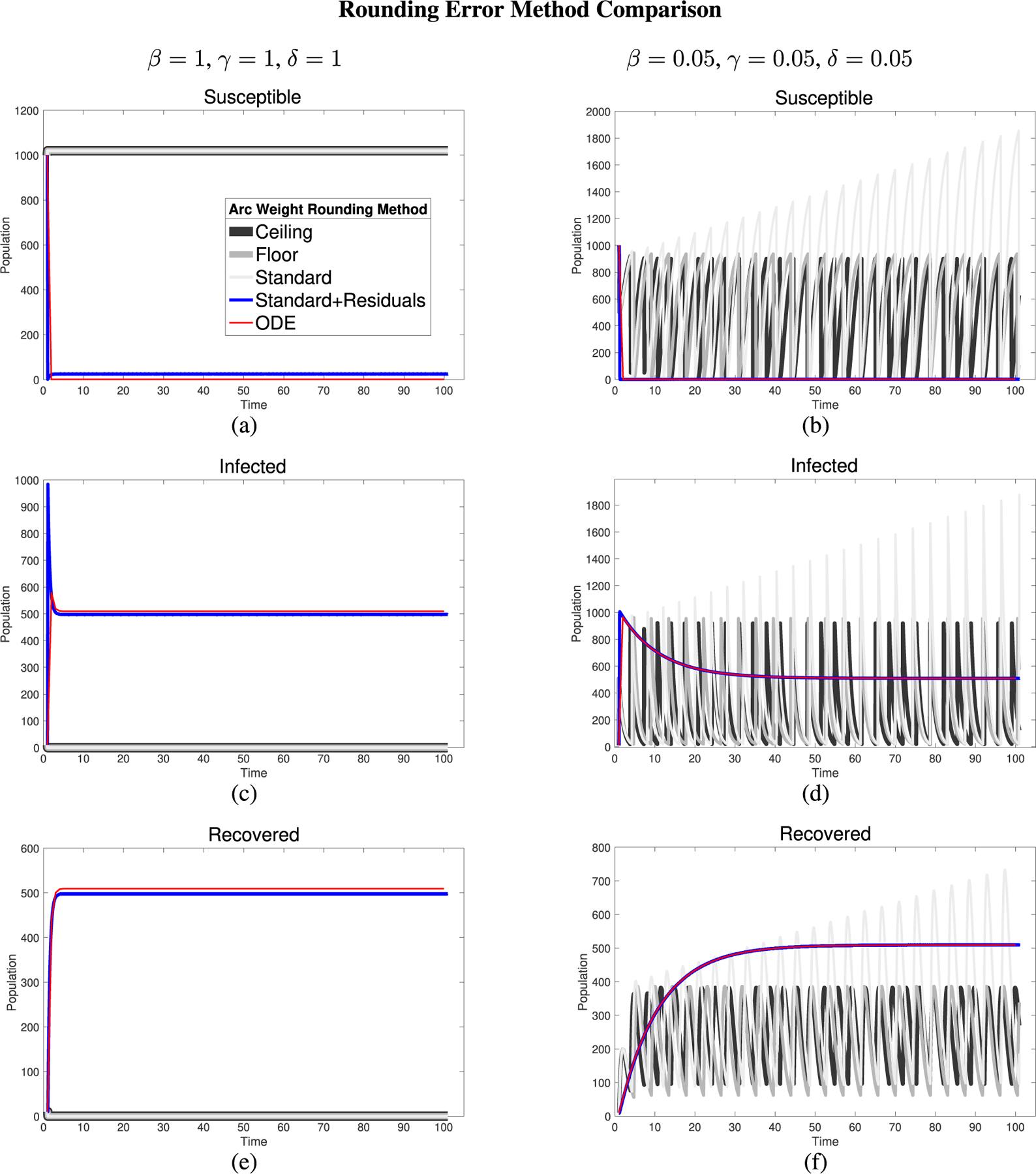
Comparison of rounding method performance on simulated data. Left column has β=1,γ=1,δ=1. Right column has β=0.05,γ=0.05,δ=0.05. Susceptible, infected, and recovered populations are shown on top, middle, and bottom rows, respectively. The legend applies to all figures. All rounding method comparisons were conducted at a time step of 20 PN time steps per unit time per 1 ODE time interval.

In all of the Figure 5‘s subfigures, the PN time steps per unit time (τ) is set to 20. Initially, when viewing Figures 5a, 5c, and 5e, we can see that the non-residual rounding methods are unable to capture the dynamics of the ODE at all. Granted, this is for the most extreme case for the parameter values, but it is important that the PN model is capable of capturing the dynamics for all values [0,1] for β,γ, and δ. When looking at more biologically plausible values of β,γ,δ=0.05 in Figures 5b, 5d, and 5f we can see that the nature of PN firing only when transitions are enabled combined with the extreme value situation as laid out in section II-D1, the dynamics behave like a 2-cycle. Without the extra logic statements laid out in section II-D1, the dynamics are even less biologically plausible, though, with no new infected even with high levels of infected and susceptible, simply with S<βSI.

### B. RESCALING OF BASE TIME STEP IN GPENSIM MODEL

The PN model, as seen in Figure 3, with different PN time steps per unit time (τ) are compared to the ODE model Equations 1–3 with the same parameters values using RRMSE. The parameter grid chosen was a linearly spaced grid of size ten between [0,1] for parameters β and γ. These are the x-axis and y-axis, respectively, for all of the sub-figures in Figures 6–27. Then for δ there is a logarithmic spaced grid of size five between [0,1] going from the top to bottom row of Figures 6–27.

**FIGURE 6.**
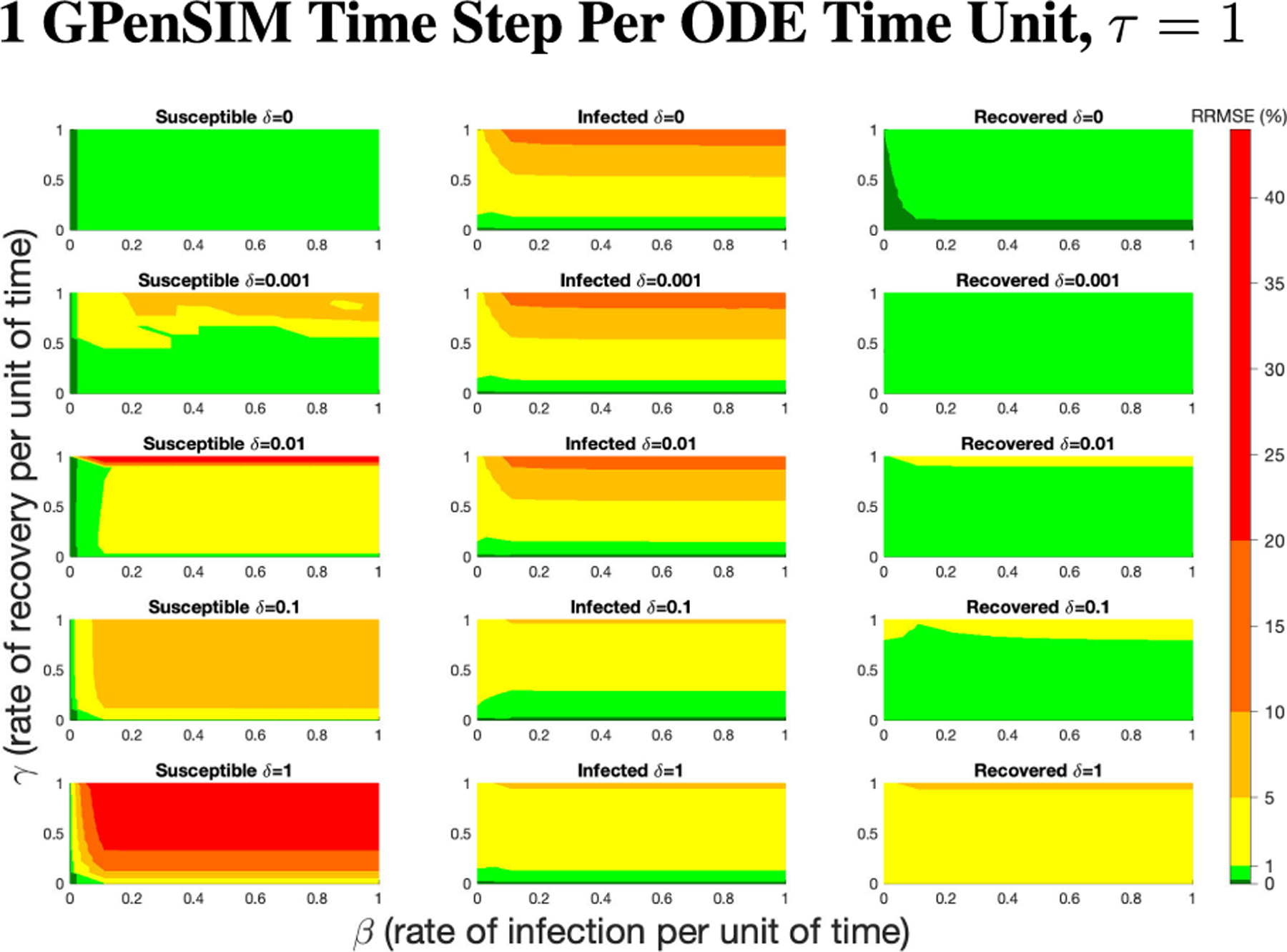
RRMSE percentage across parameter ranges of [0, 1] for each respective parameter with γ the y-axis of each subfigure, β the x-axis of each subfigure, and δ set at a different fixed value for each subfigure. PN Time Step Per Unit of Time parameter τ=1. Note that red is RRMSE ≤ 44% (43.75% being the max observed RRMSE across all simulations), dark orange is RRMSE ≤ 20%, light orange is RRMSE ≤ 10%, yellow is RRMSE ≤ 5%, light green is RRMSE ≤ 1%, and dark green is RRMSE ≤ .1%.

When the τ=1, in Figure 6 the RRMSE performance is relatively poor, especially for higher values of β,γ, and δ. This low τ value displays the problem of comparing a discrete versus a continuous time system with large swings in population happening instantly at the time step, not allowing the dynamics of the PN to come close to matching that of an ODE continuous system. These sharp dynamics combined with the additional firing mechanisms of PN means the RRMSE values produced are relatively large.

**FIGURE 7.**
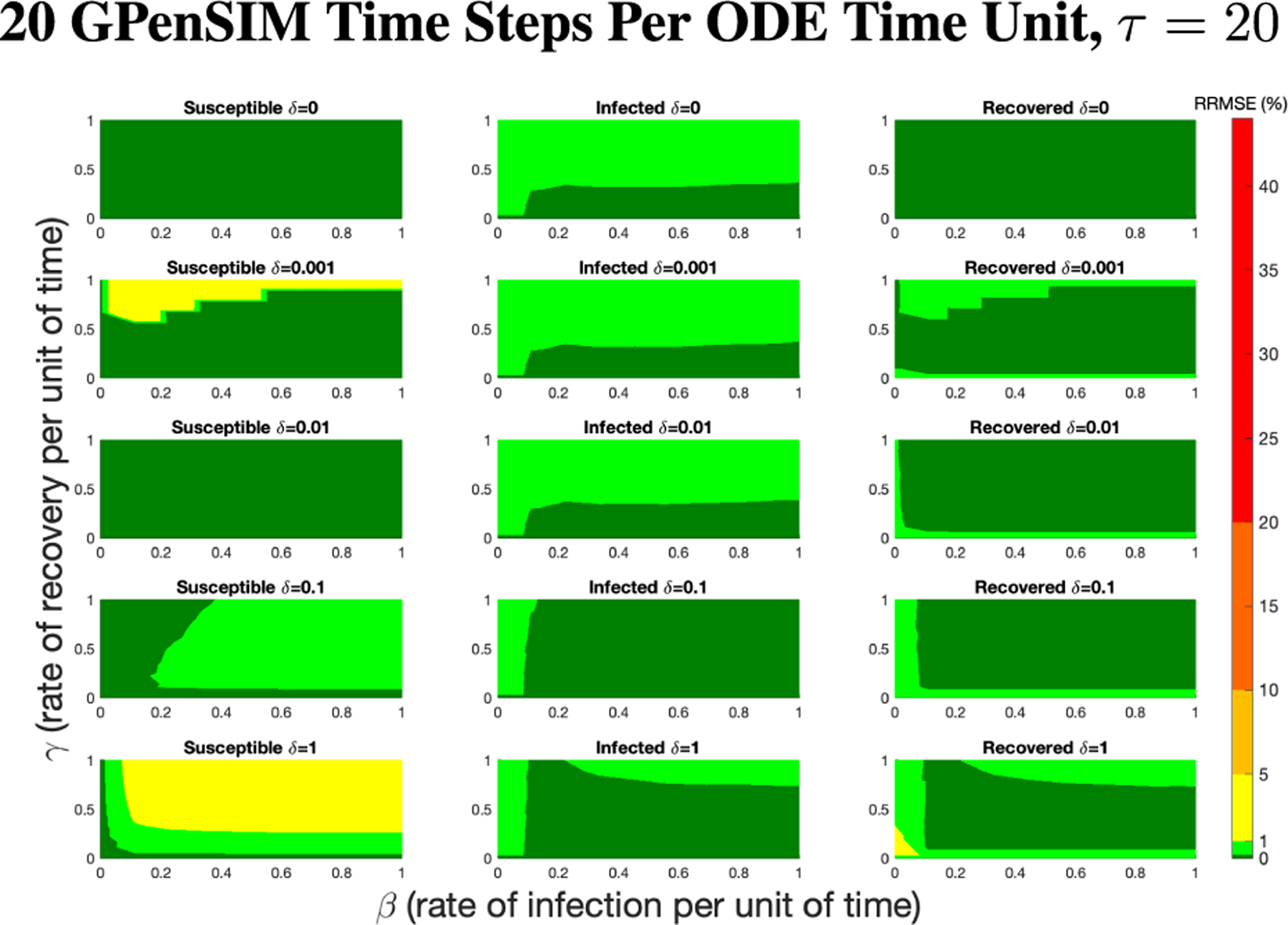
RRMSE percentage across parameter ranges of [0, 1] for each respective parameter with γ the y-axis of each subfigure, β the x-axis of each subfigure, and δ set at a different fixed value for each subfigure. PN Time Step Per Unit of Time parameter τ=20. Note that yellow is RRMSE ≤ 5%, light green is RRMSE ≤ 1%, and dark green is RRMSE ≤ 0.1%.

Figure 7 shows the vast improvement in RRMSE with utilizing higher PN time steps per unit time. With this one change, the maximum RRMSE becomes approximately 4.3, and the visual improvement at extreme values is considerable.

**FIGURE 8.**
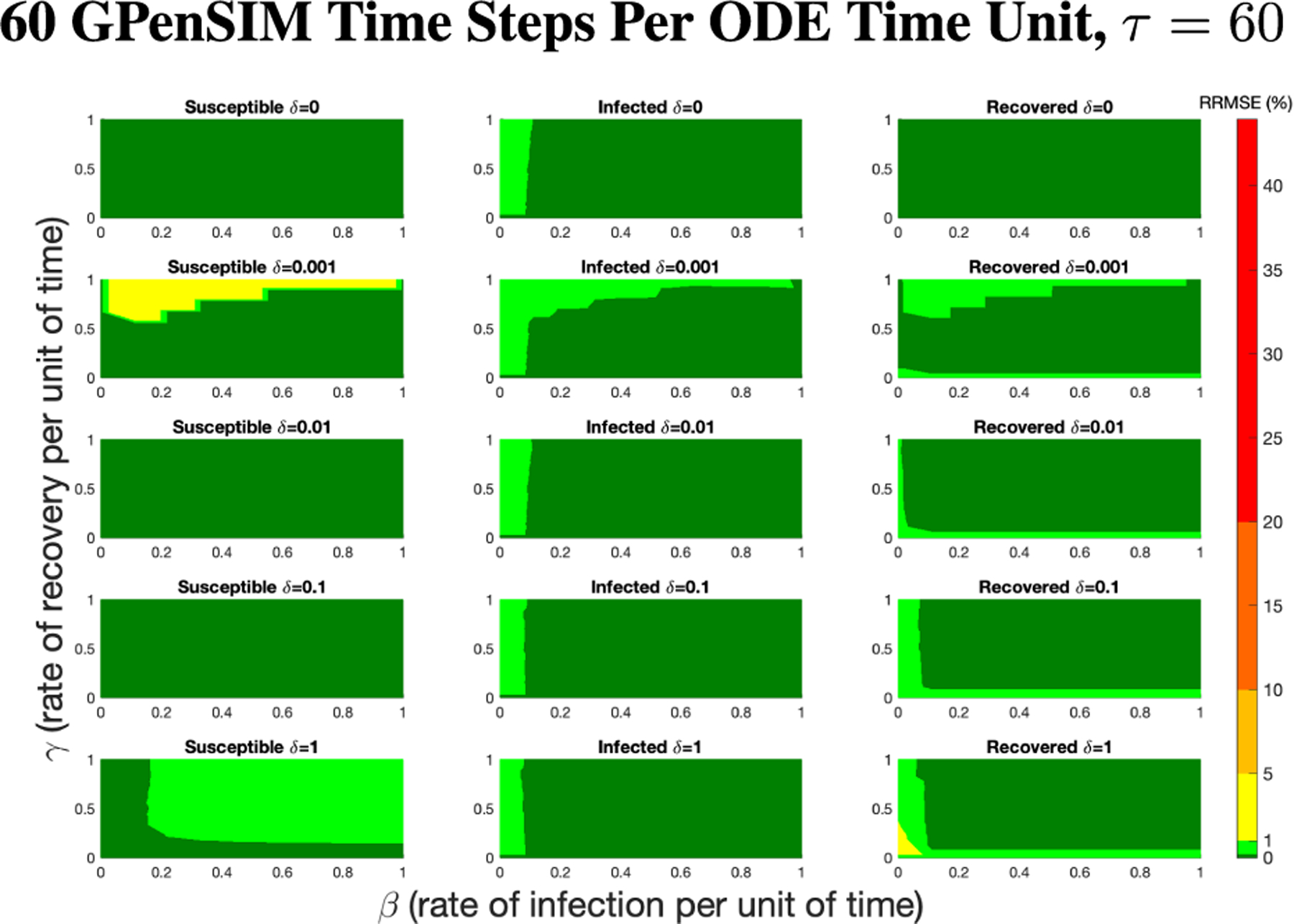
RRMSE percentage across parameter ranges of [0, 1] for each respective parameter with γ the y-axis of each subfigure, β the x-axis of each subfigure, and δ set at a different fixed value for each subfigure. PN Time Step Per Unit of Time parameter τ=60. Note that yellow is RRMSE ≤ 5%, light green is RRMSE ≤ 1 %, and dark green is RRMSE ≤ 0.1%.

**FIGURE 9.**
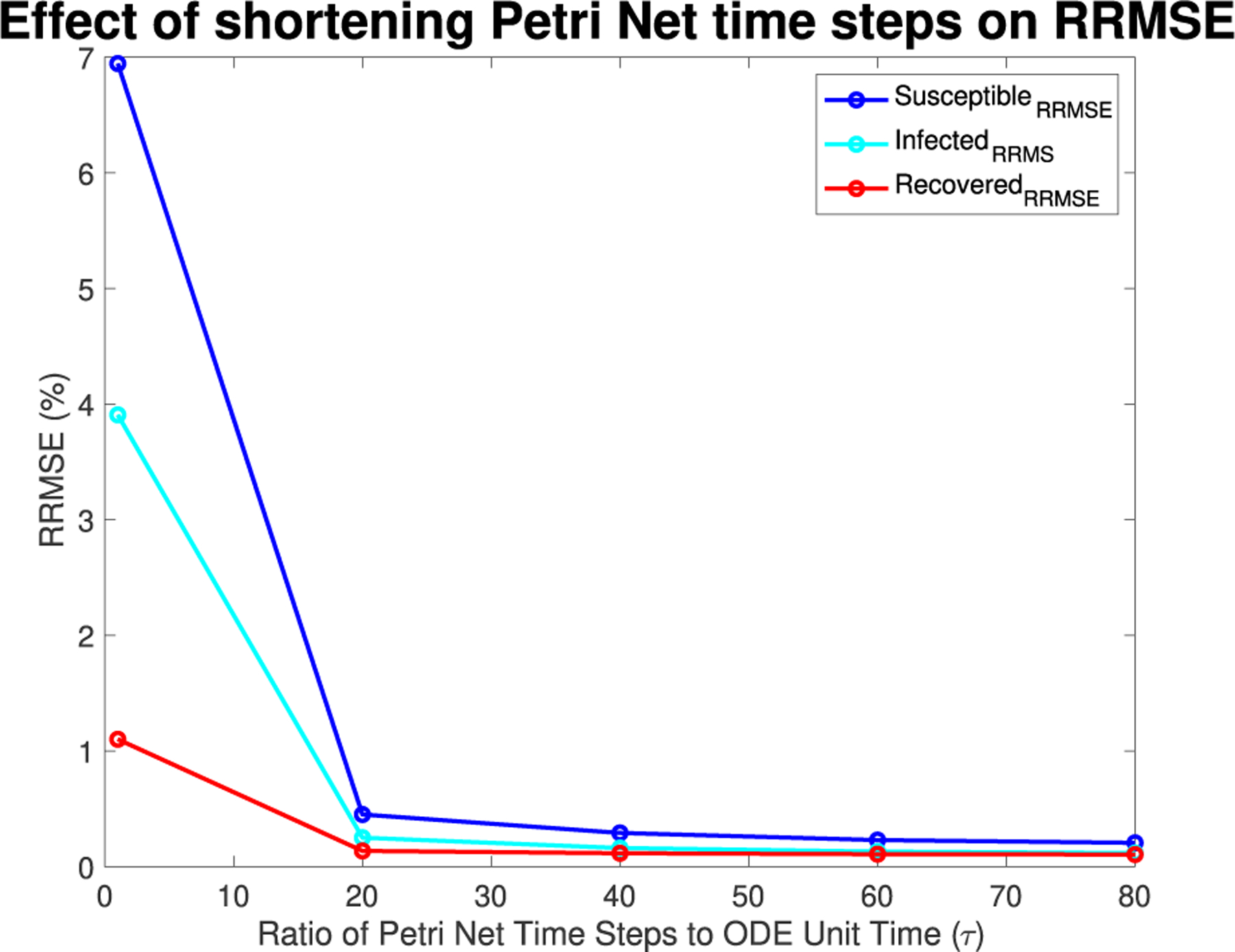
Mean RRMSE in percentage across parameter ranges of γ range of [0,1] with 10 linearly spaced points, β range of [0,1] with 10 linearly spaced points, and δ range of [0,1] with 5 logarithmically spaced points.

As τ increases from one to eighty, there is an overall reduction of RRMSE with each subsequent increase of the PN time steps per unit time, which can be more clearly seen in Figure 9. This can be seen for each run in Figures 6,7,8 and Figures 26,27 in the appendix.

### C. POPULATION SCALING FOR GPENSIM AND SPIKE MODELS

The stochastic Petri net system used in Spike and the dynamic arc weight Petri net system used in GPenSIM both use discrete time and rounding of arcs weights to whole numbers. As such, it was theorized that scaling the initial populations and descaling after the simulations have occurred would reduce overall RRMSE [16].

For GPenSIM we started with a τ=60 since this setting lead to small RRMSEs overall and lower computation time. Figure 12 shows the results of rescaling the population size by a factor of 4.

In Figure 12, all of the parameter value combinations produce RRMSE less than 1%, with the majority of the values being less than 0.1%. While it depends on the application, we consider this to be an acceptable level for the Petri net model.

When fitting parameters to a Petri net model using data and wanting to maintain a low RRMSE in relation to a corresponding ODE model, finding the minimum population scalar and τ value to minimize computation time is an important and practical problem. Figure 15 shows what this process can look like across a single parameter set (β=0.1,γ=0.5,δ=0.001). From Figure 15 we can see that if we are focused on the all populations maintaining RRMSE below 0.1%, then a population scalar of 1 and τ=40 would suffice. An algorithm could then be developed for optimizing conditions over the parameter ranges to minimize fitting computation time, but this is a subject for future work.

For Spike, we can not directly alter the τ value, but we can directly alter the initial population size. Given Spike’s relatively low computation time for the SPN, we were able to raise the population scalar to much higher values, starting at one and going up by order of magnitude each time. We stopped when we reached a level of less than 1% RRMSE across all parameter values for the direct and tau-leaping method for SPNs. Though Spike demonstrates significantly better computational efficiency relative to GPenSIM, we limited all SPN simulations in Spike to 500 trials. This ensures manageable computation times while maintaining the accuracy and statistical robustness needed for meaningful results.

In Figures 16–18 we see the RRMSE drop as the population scalar increases, to a level where for all parameter values are less than 1% RRMSE with a population scalar of 100 for the direct method. Beyond a population scalar of 100, the regions of less than 0.1% RRMSE remain nearly identical when the population scalar is raised by an order of magnitude. This likely indicates that either an asymptotic convergence of the means is occurring or some other factor is limiting the RRMSE from decreasing further.

**FIGURE 10.**
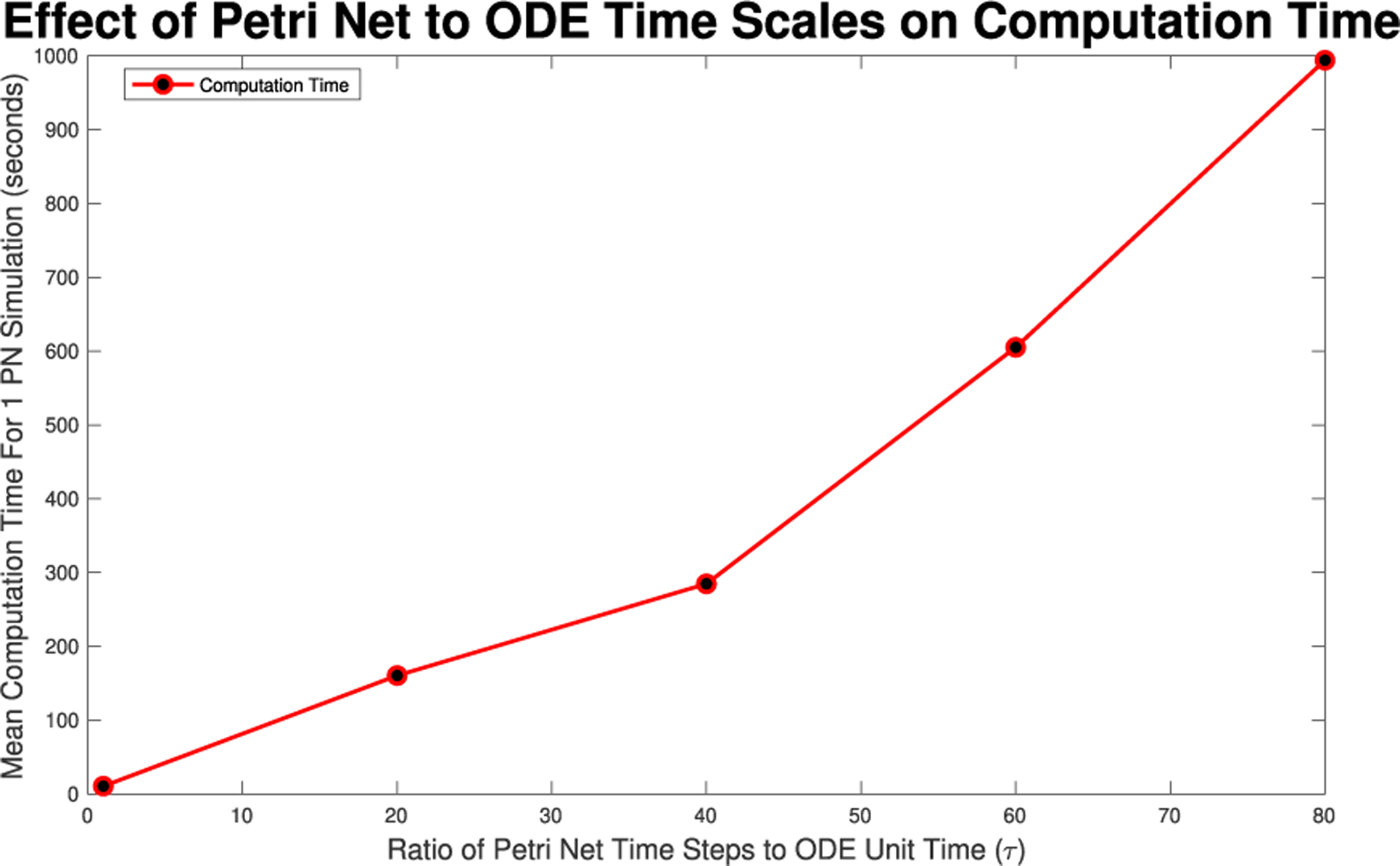
Mean computation time for one dynamic arc weight PN model run in GPenSIM for various times steps as seen in Figures 6, 7, 8, 26, and 27.

**FIGURE 12.**
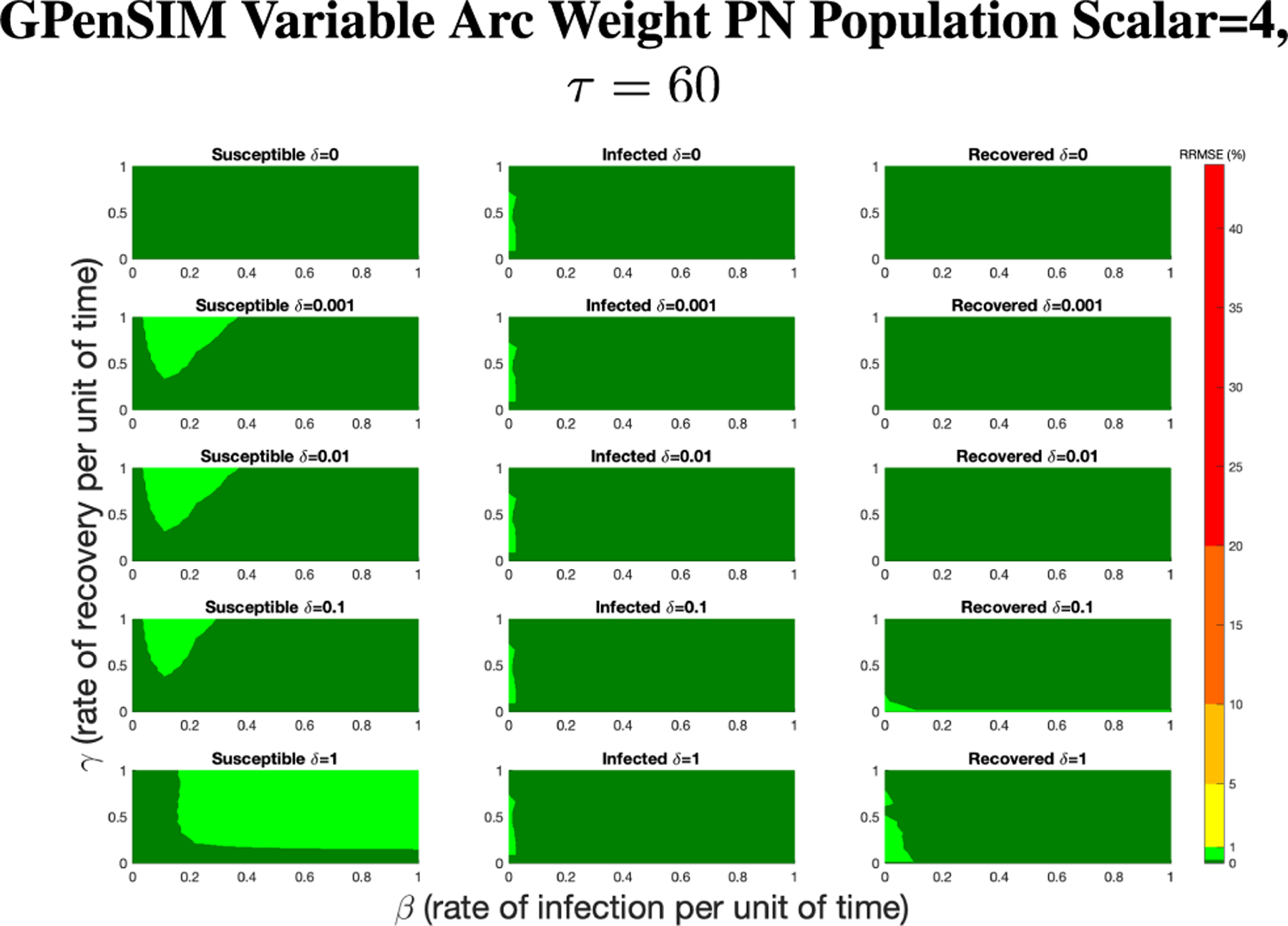
RRMSE percentage across parameter ranges of [0, 1] for each respective parameter with γ the y-axis of each subfigure, β the x-axis of each subfigure, and δ set at a different fixed value for each subfigure. PN Time Step Per Unit of Time parameter τ=60. Note that light green is RRMSE ≤ 1%, and dark green is RRMSE ≤ 0.1%.

**FIGURE 14.**
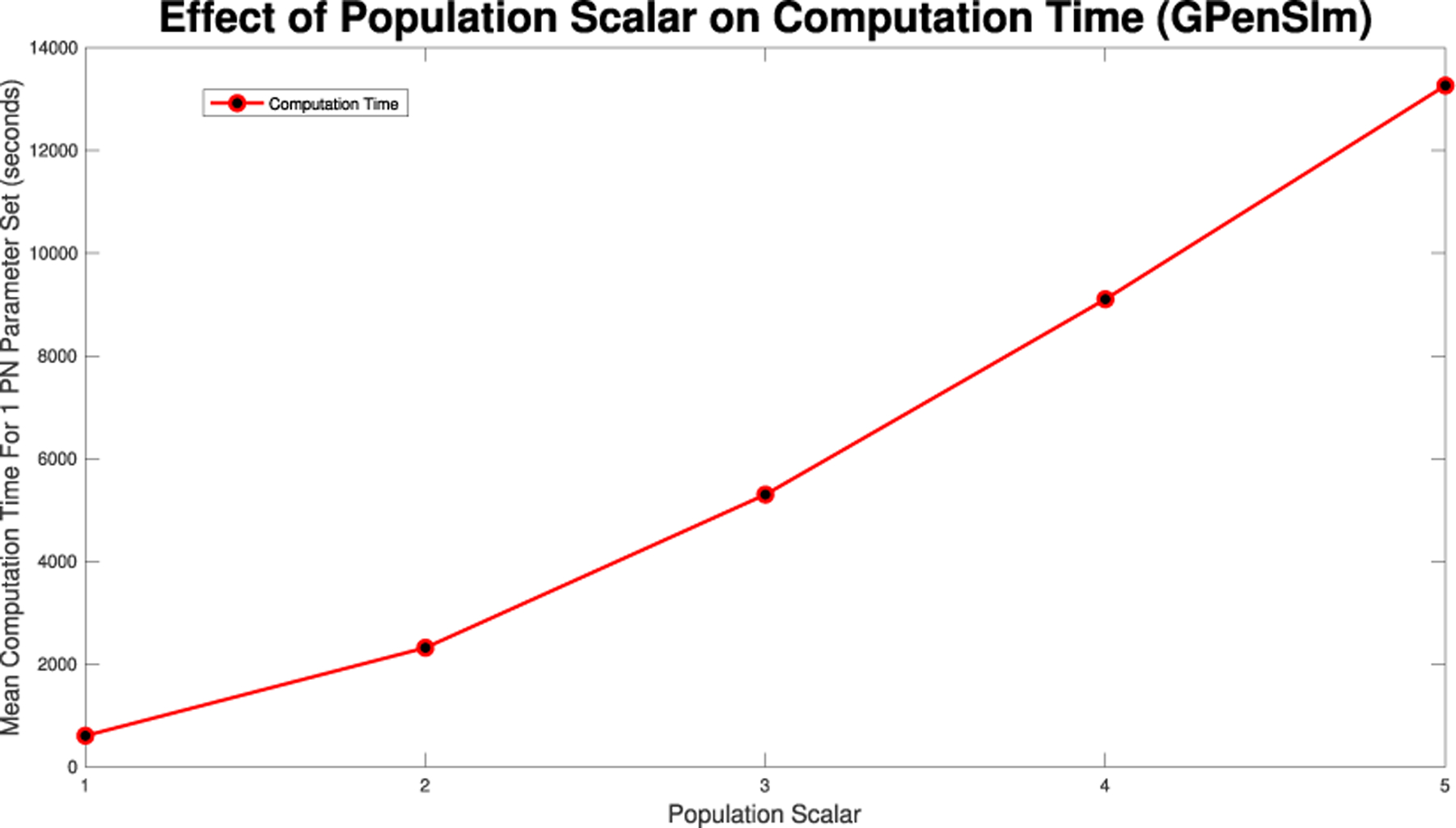
Mean computation time for one dynamic arc weight PN model run in GPenSIM for increasing population scalars as seen in Figures 8,11,12, and 13.

We note that the computing demand for single runs of these simulations are low enough to run on a laptop and do not require a supercomputer cluster. However, when running large sets of parameters, with large population scalars and large τ values parallelization, a supercomputer, or both, will allow for the simulations to be run in a reasonable time. For Figures 10,14,19, and 24 the values were found while being run in MATLAB 2024b, using GPenSIM v. 10 software and Spike v1.6 respectively, on a computer with a 2.3 GHz 8-Core processor, with 64 GB of memory. These models were also run on the Arizona State University SOL supercomputer, where even when allocated four cores and 32 GB of memory, the models only had a maximum memory used value of 5.6 GB.

## IV. DISCUSSION AND CONCLUSION

Petri nets are increasingly used to model the dynamics of infectious disease spread, building on a deep literature of ODE models. However, few studies have examined the numerical equivalency of Petri net models to reference ODE systems, especially beyond the most basic SIR model. In this paper, we introduced a novel PN implementation of SIR-type models using a discrete-time, variable arc weight framework in the software package GPenSIM. We explored the numerical equivalency of this variable arc weight model to an SIRS ODE model, and we found that several choices in implementation are important for matching the ODE dynamics, including the rounding method used for non-integer arc weights and rescaling of the time step for large arc weight values. We also compared the behavior of a continuous-time Markov Chain PN using the software package Spike. For both types of PN, we found that rescaling the population size led to major improvements in the relative root mean square error comparing the PN trajectories to the reference ODE behavior. This indicates that PN models do not necessarily generate identical dynamics to their ODE counterparts, and that prior numerical studies are an important tool for ensuring similarity.

Variable arc weight Petri nets using GPenSIM software represent a useful technical breakthrough in Petri net modeling by allowing dynamic adjustments to the arc weights based on real-time conditions or external state changes rather than static values. This numerical flexibility is complemented by recent theoretical advancements. For instance, Reckell et al. [20] demonstrated that the basic reproduction number $\left(R_{0}\right)$ can be rigorously derived for Petri net models using a Next-Generation Matrix approach. Together, these findings ensure that Petri net models are grounded in the same fundamental epidemiological metrics as their ODE counterparts while offering distinct modeling advantages. GPenSIM’s ability to incorporate variable arc weights yields an additional PN modeling option beyond the CTMC framework. Furthermore, the flexibility of this framework extends beyond the SIRS model presented here. The modular nature of Petri nets allows for the straightforward addition of compartments, such as an Exposed (E) state for SEIRS models, or stratification by age and risk groups. As explored in Reckell et al. [20], this framework supports complex SEIR models, demonstrating that metrics like $R_{0}$ remain derivable even with variable arc weights. This adaptability makes the numerical convergence findings presented here relevant for a broad class of compartmental epidemiological models.

**FIGURE 15.**
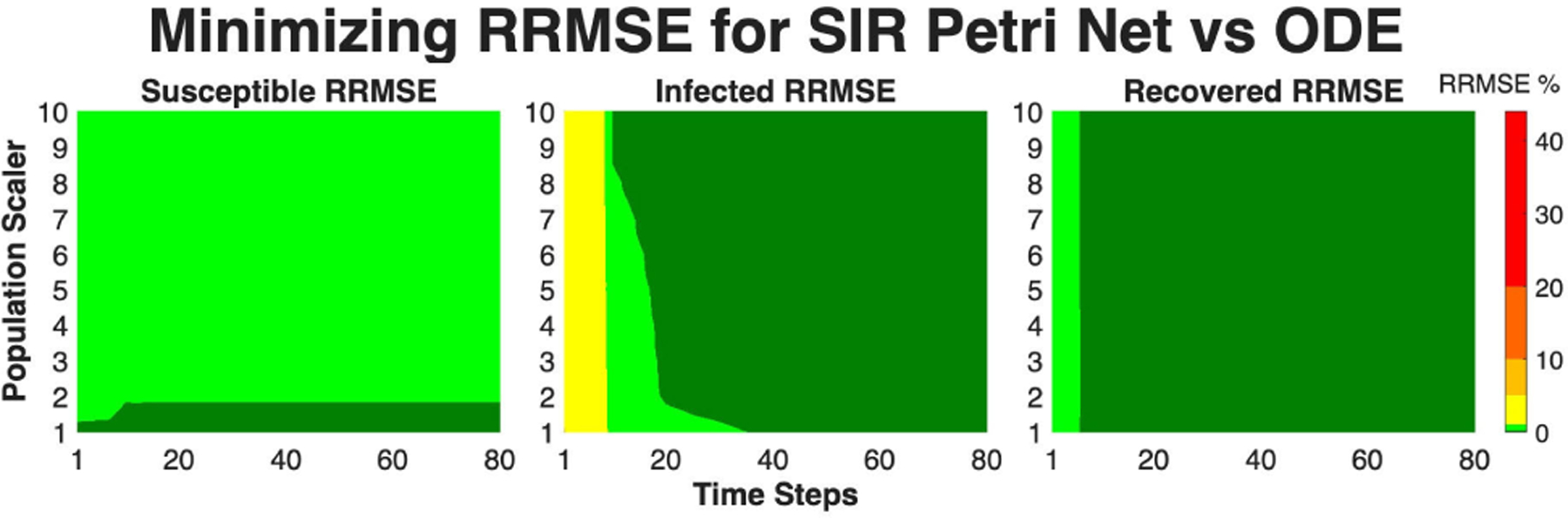
RRMSE percentage for a fixed parameter set (β=0.1,γ=0.5,δ=0.001) with population scalar on the y-axis and time steps (τ) on the x-axis. Note that light green is RRMSE ≤ 1%, and dark green is RRMSE ≤ 0.1%.

**FIGURE 16.**
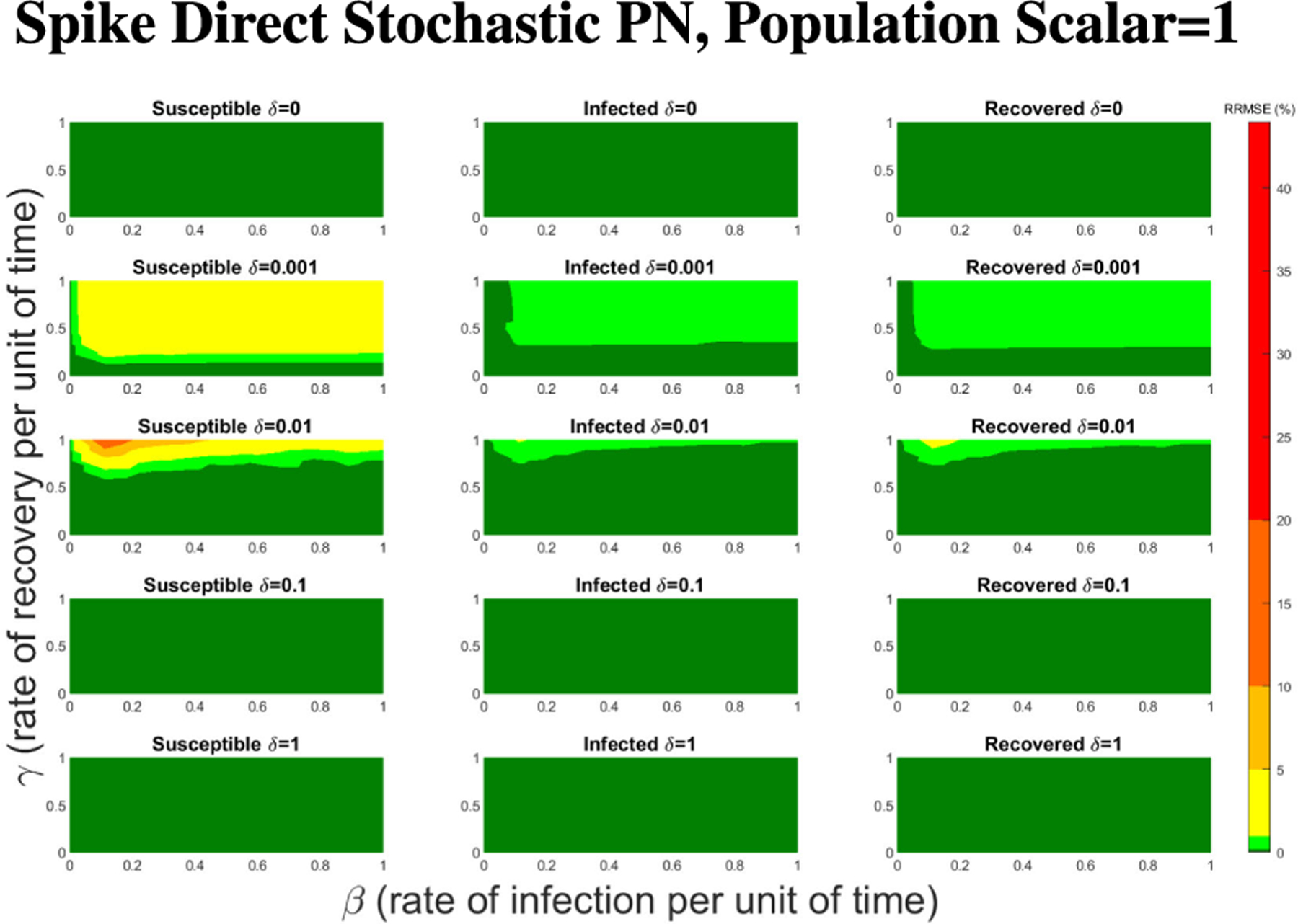
RRMSE percentage across parameter ranges of [0, 1] for each respective parameter with γ the y-axis of each subfigure, β the x-axis of each subfigure, and δ set at a different fixed value for each subfigure. Note that red is RRMSE ≤ 44% (43.75% being the max observed RRMSE across all simulations), dark orange is RRMSE ≤ 20%, light orange is RRMSE ≤ 10%, yellow is RRMSE ≤ 5%, light green is RRMSE ≤ 1%, and dark green is RRMSE ≤ .1%.

**FIGURE 17.**
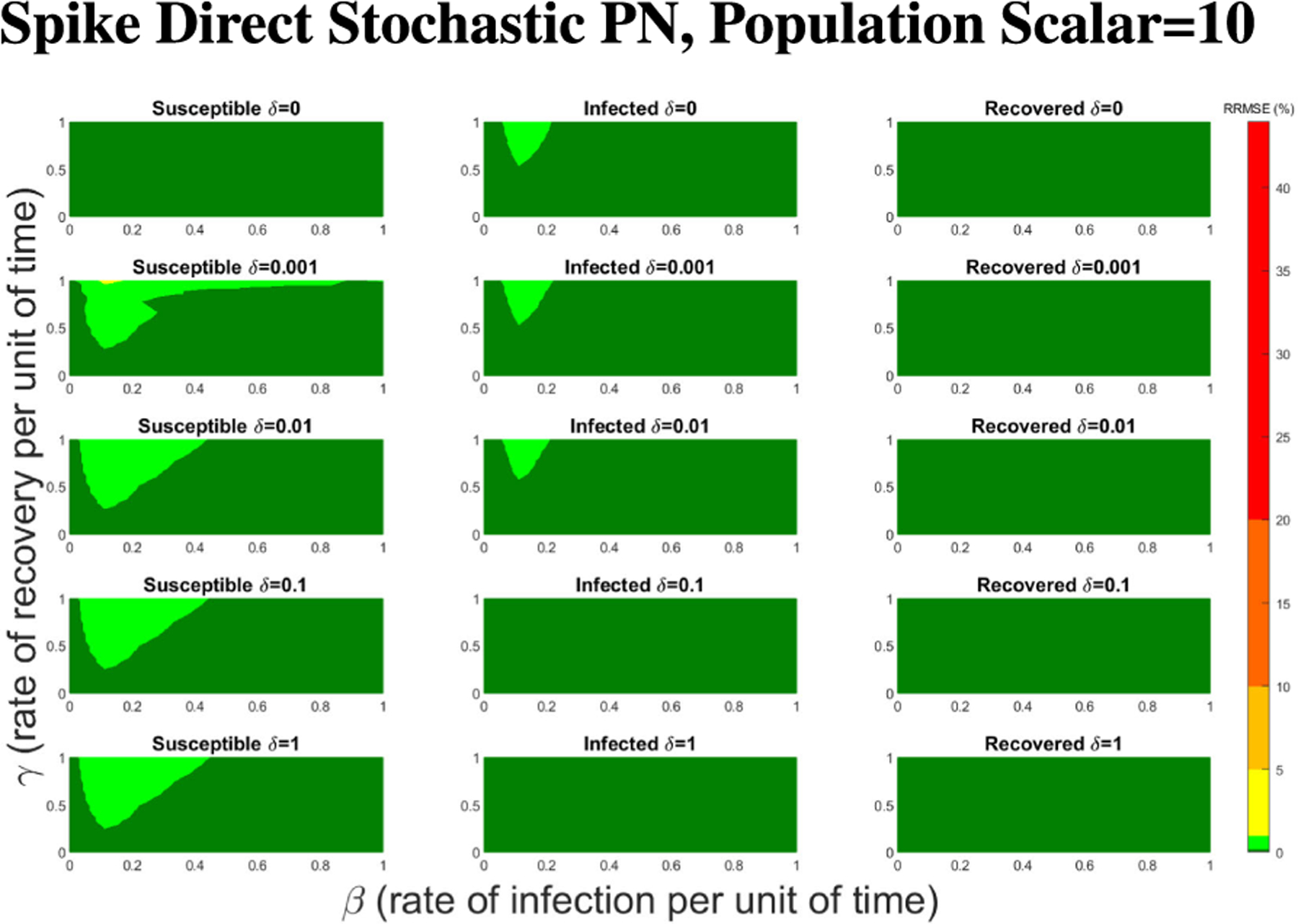
RRMSE percentage across parameter ranges of [0, 1] for each respective parameter with γ the y-axis of each subfigure, β the x-axis of each subfigure, and δ set at a different fixed value for each subfigure. Note that yellow is RRMSE ≤ 5%, light green is RRMSE ≤ 1%, and dark green is RRMSE ≤ .1%.

**FIGURE 18.**
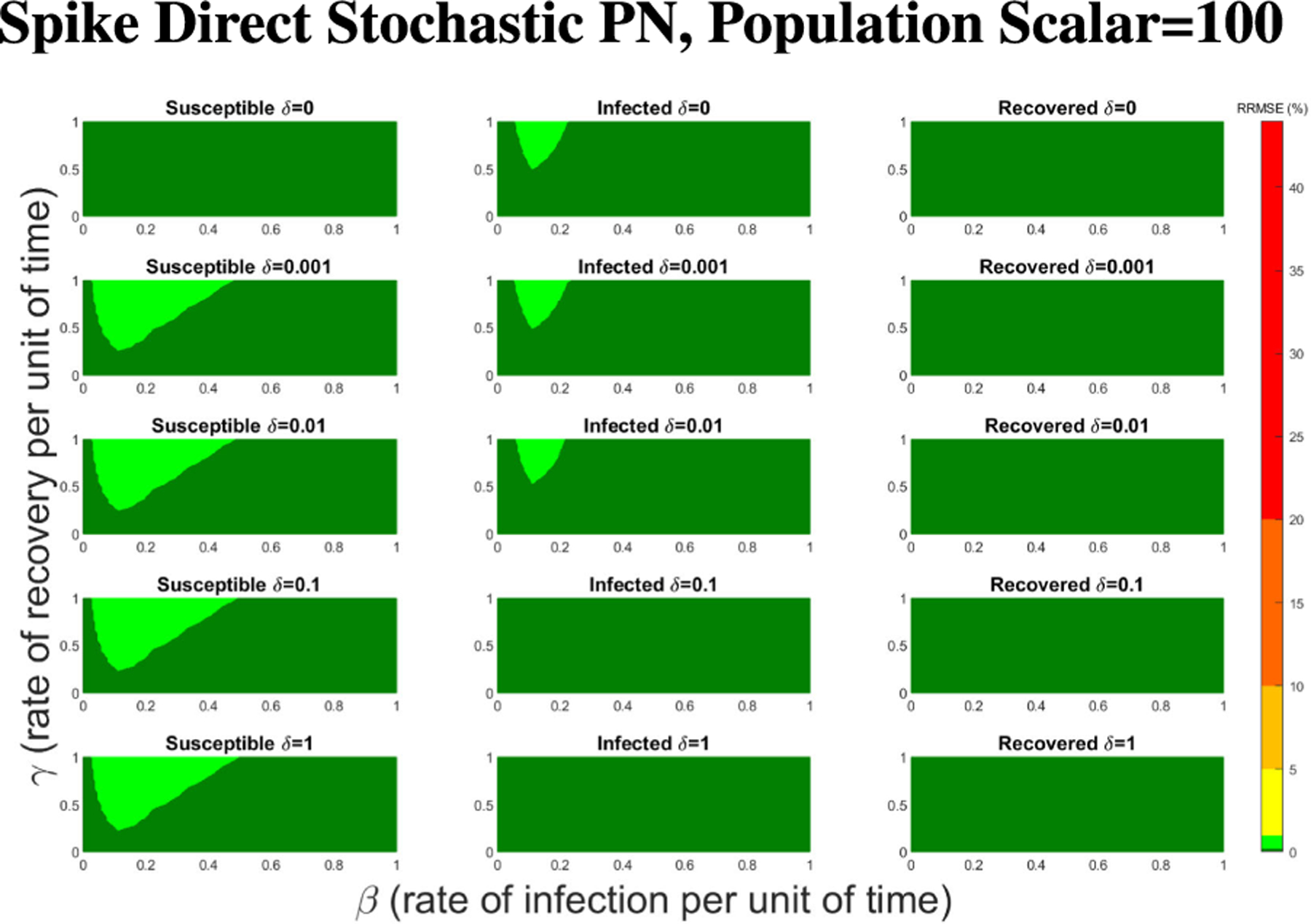
RRMSE percentage across parameter ranges of [0, 1] for each respective parameter with γ the y-axis of each subfigure, β the x-axis of each subfigure, and δ set at a different fixed value for each subfigure. Note that light green is RRMSE ≤ 1%, and dark green is RRMSE ≤ .1%.

**FIGURE 19.**
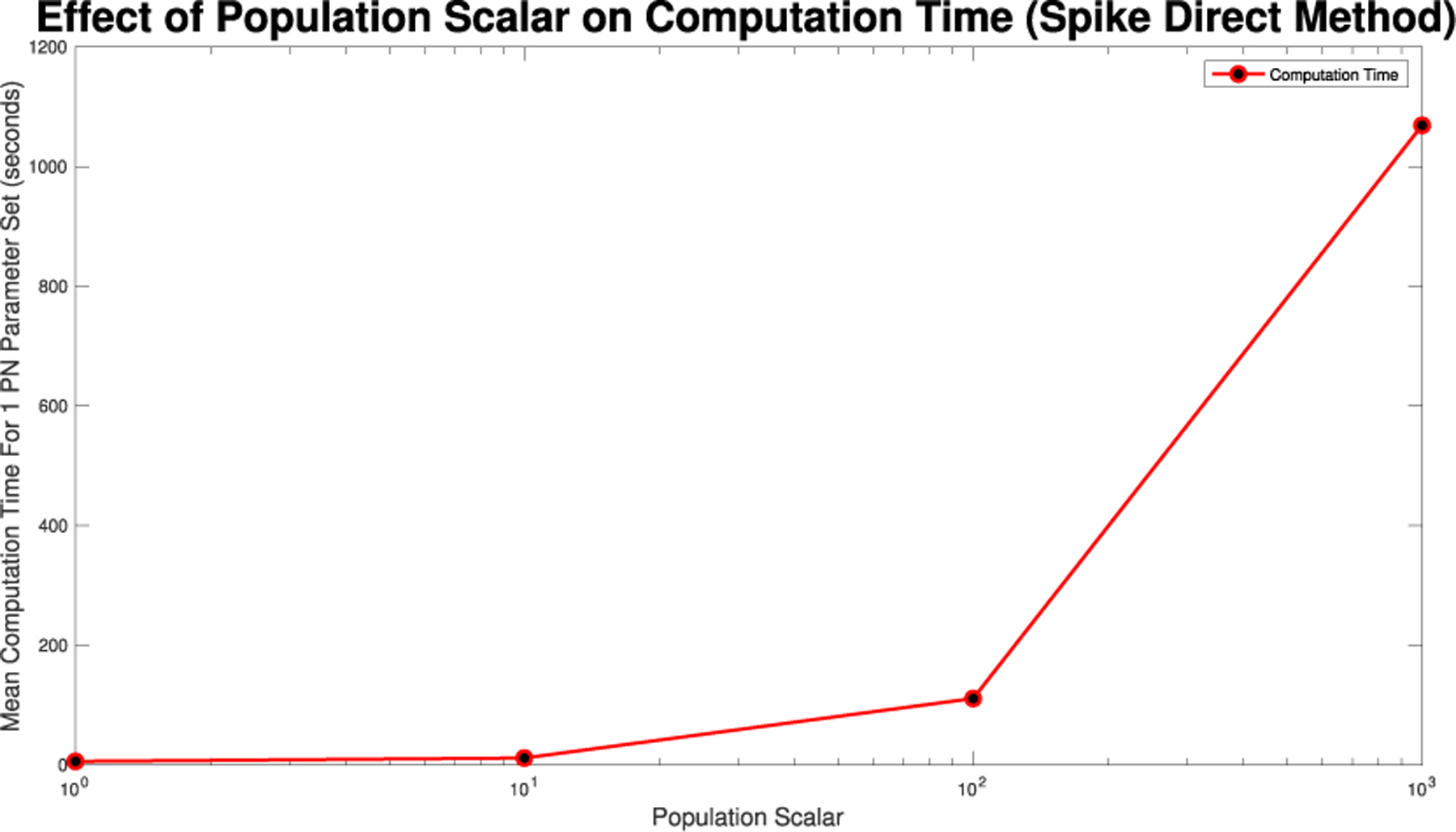
Mean computation time for one dynamic arc weight PN model run in Spike using the Direct method for increasing population scalars as seen in Figures 16–18.

**FIGURE 20.**
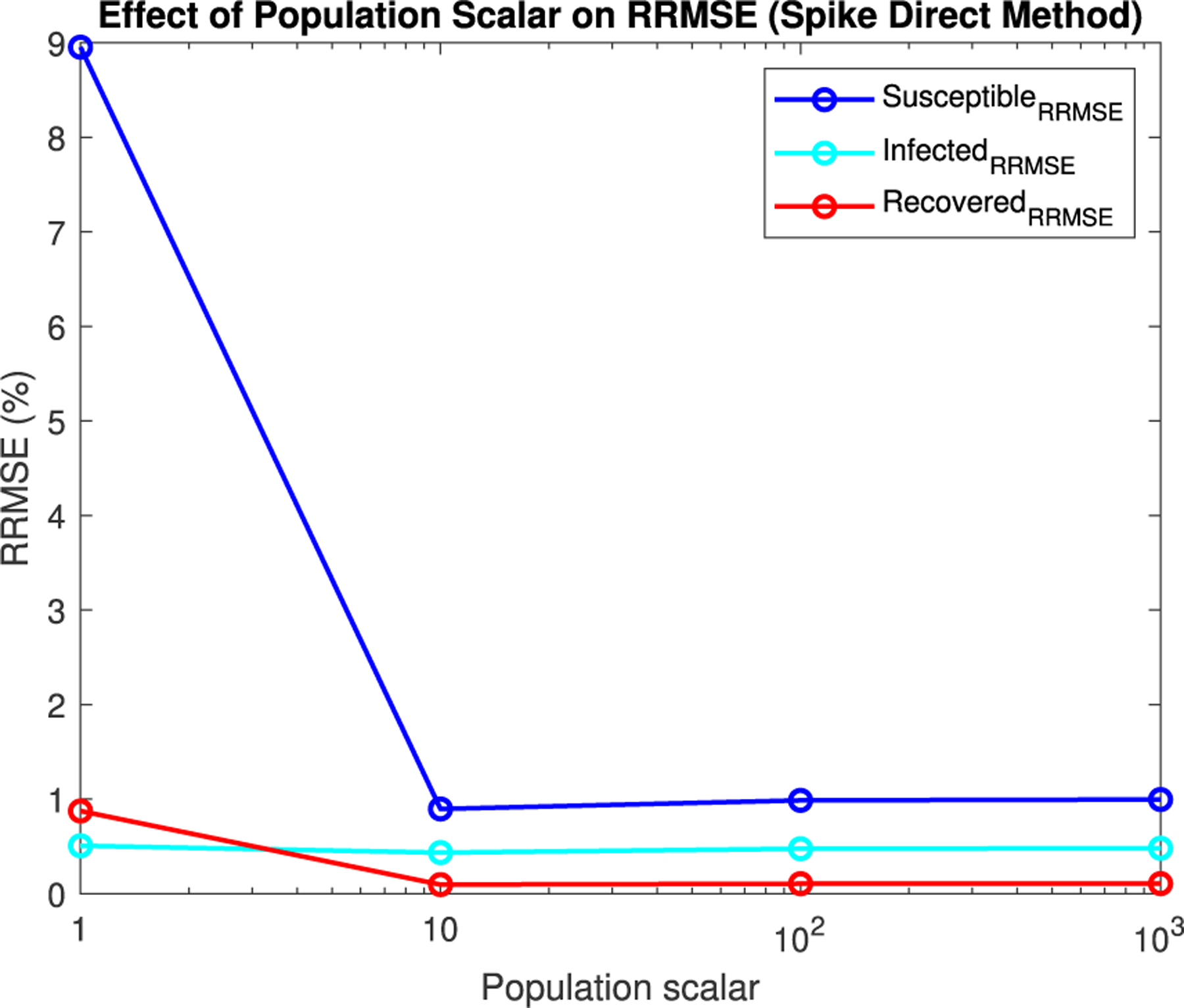
Mean RRMSE in percentage for selected parameters values, γ=0.5320,β=0.0500, and δ=0.0025. These values were not included in the parameter grid used in the main experiment.

**FIGURE 24.**
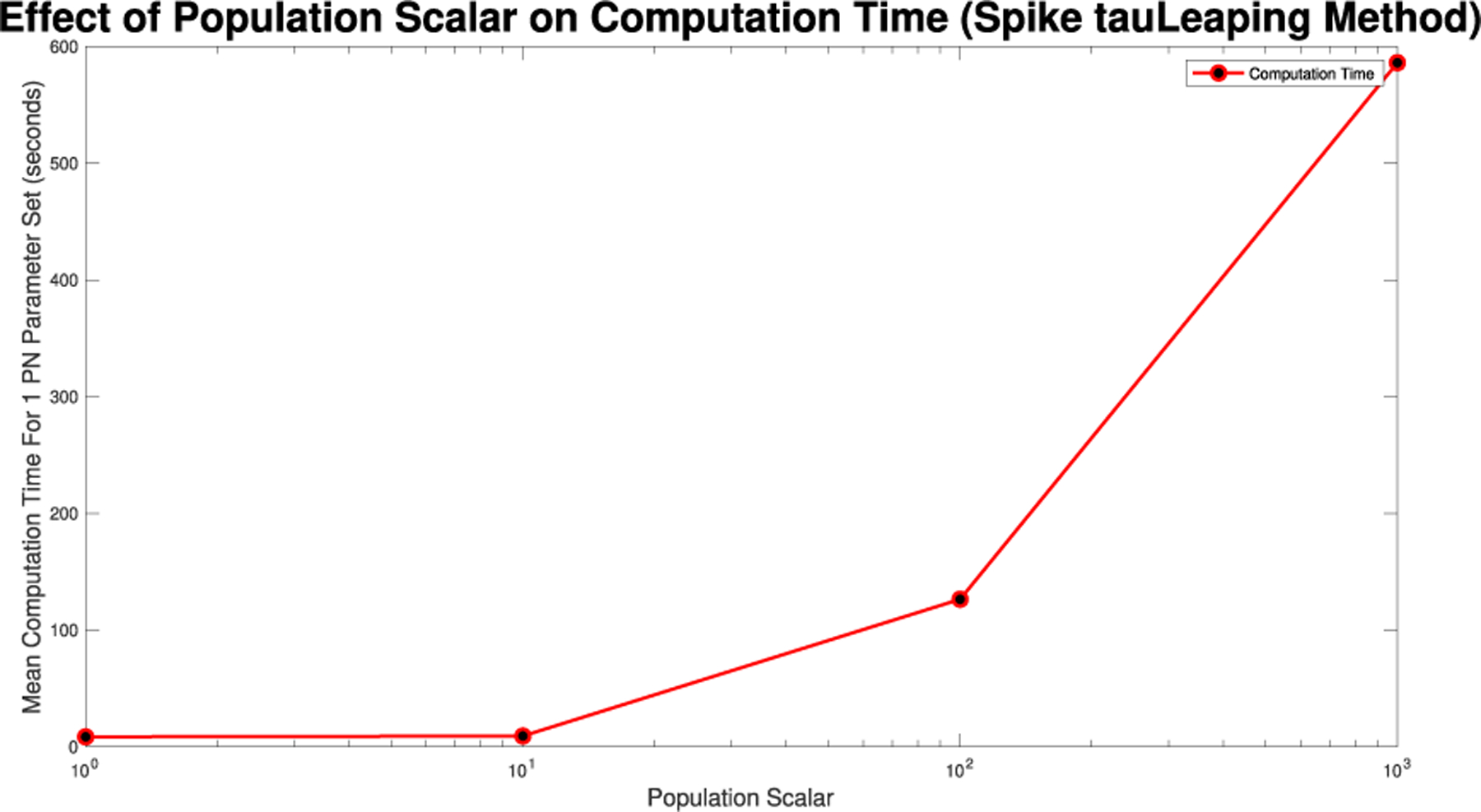
Mean computation time for one dynamic arc weight PN model run in Spike using the tauLeaping method for increasing population scalars as seen in Figures 21–23.

**FIGURE 25.**
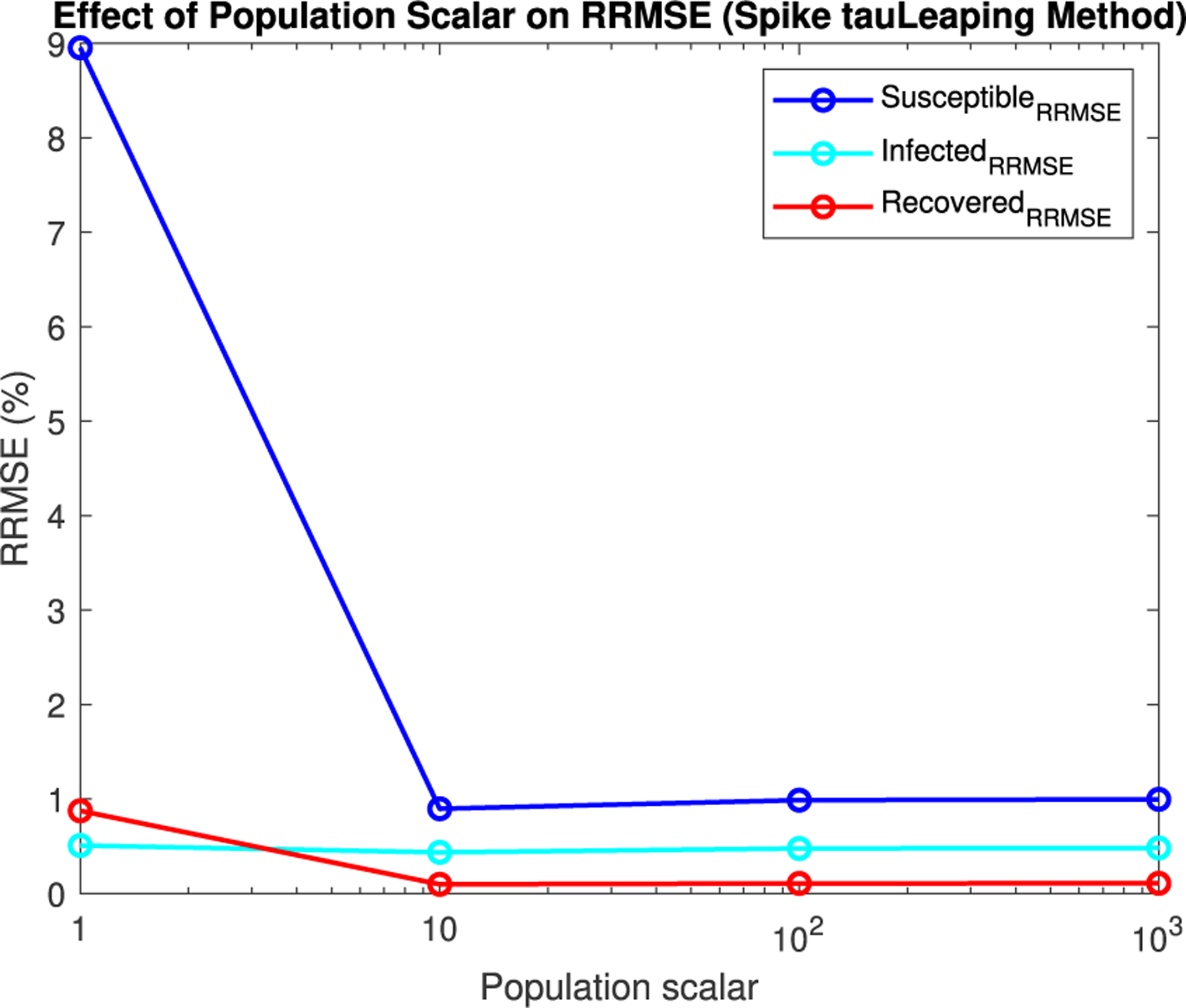
Mean RRMSE in percentage for selected parameters values, γ=0.5320,β=0.0500, and δ=0.0025. These values were not included in the parameter grid used in the main experiment.

**FIGURE 26.**
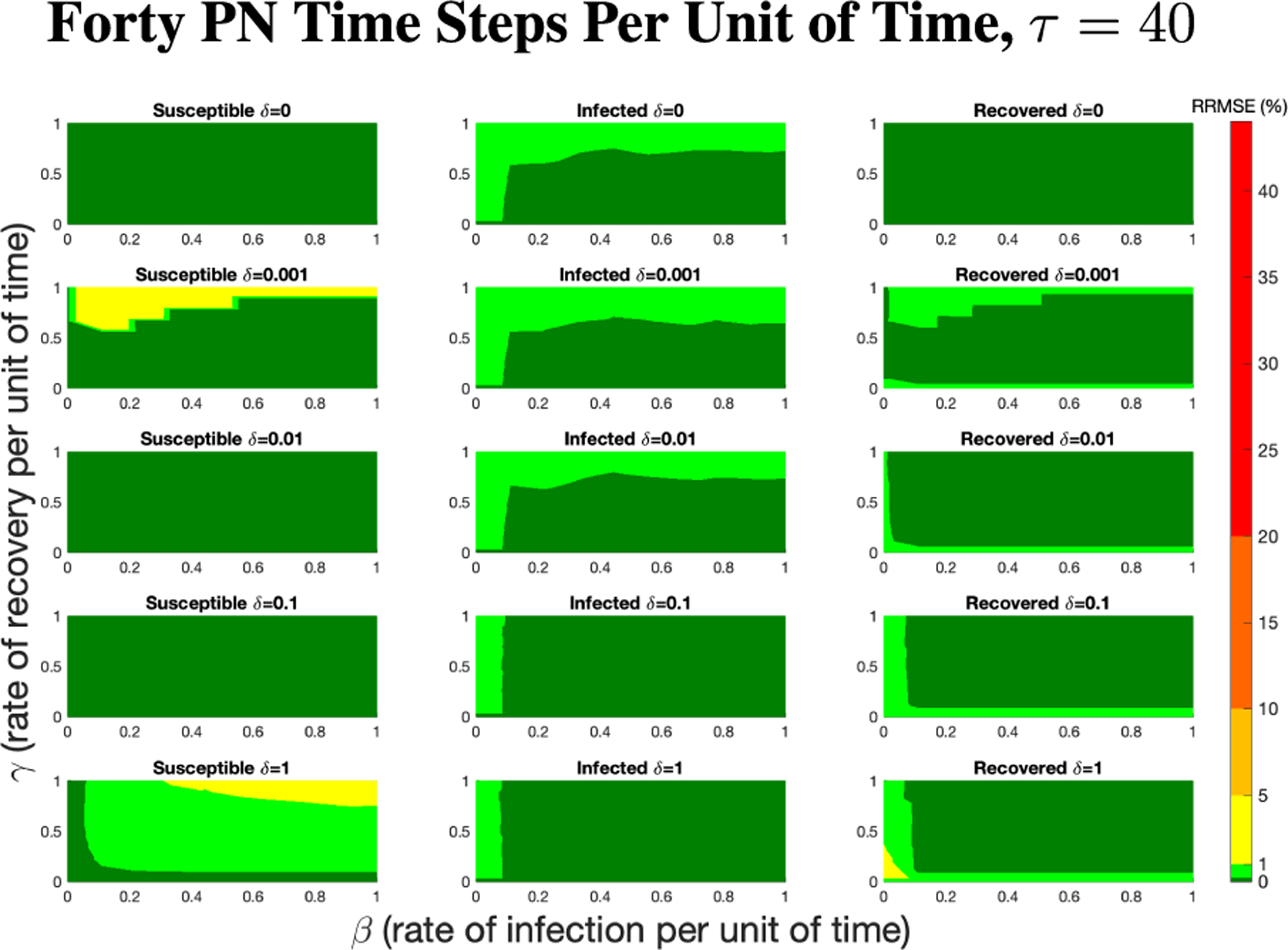
RRMSE percentage across parameter ranges of [0, 1] for each respective parameter with γ the y-axis of each subfigure, β the x-axis of each subfigure, and δ set at a different fixed value for each subfigure. PN Time Step Per Unit of Time parameter τ=40. Note that yellow is RRMSE ≤ 5%, light green is RRMSE ≤ 1%, and dark green is RRMSE ≤ 0.1%.

**FIGURE 27.**
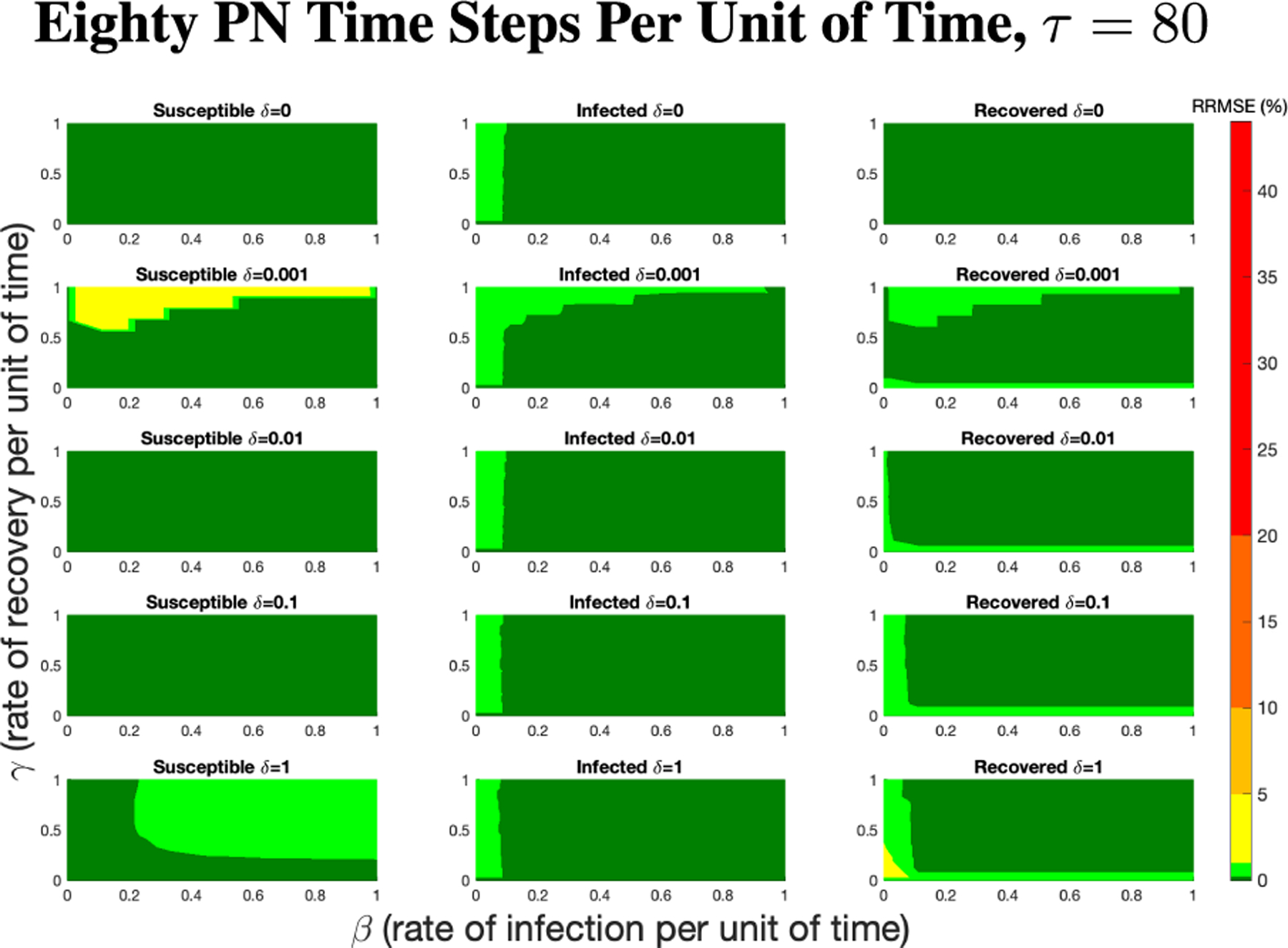
RRMSE percentage across parameter ranges of [0, 1] for each respective parameter with γ the y-axis of each subfigure, β the x-axis of each subfigure, and δ set at a different fixed value for each subfigure. PN Time Step Per Unit of Time parameter τ=80. Note that yellow is RRMSE ≤ 5%, light green is RRMSE ≤ 1%, and dark green is RRMSE ≤ 0.1%.

Our results demonstrate that parameter optimization is key to balancing accuracy and computational cost. As shown in Figure 15, the relationship between population scalars and time steps (τ) reveals a clear optimization surface where computational efficiency can be maximized while maintaining a low RRMSE. Furthermore, the convergence behavior detailed in Figures 20 and 25 for the Spike implementation highlights that while stochastic noise is present at lower populations, both the direct and tau-leaping methods consistently converge to the ODE solution as the population scalar increases. This validates the robustness of the approach across different simulation methodologies, even when initialized with parameters outside the primary grid search as seen in Figures 20 and 25.

The demonstrated convergence has practical implications for modeling real-world disease networks, such as zoonotic spillover events or COVID-19 transmission in heterogeneous populations. In these scenarios, the ability of Petri nets to handle discrete events and individual-level stochasticity while maintaining numerical fidelity to population-level ODEs provides a robust tool for capturing dynamics that purely continuous models may miss.

The code for all of the models can be found in the GitHub https://github.com/trevorreckell/Numerical-Comparison-of-PN-vs-ODE-for-SIR. The code contains the model laid out as PN, and the corresponding ODE is defined as a function.

### LIMITATIONS AND FUTURE WORK

While our study confirms the feasibility of PN convergence to ODEs, it also highlights limitations to consider. First, the computational efficiency of the deterministic GPenSIM implementation is lower than that of the CTMC approach in Spike. Future work should investigate optimization strategies, such as modularizing the Petri net structure to enable parallel execution on high-performance computing clusters. We validated convergence for time and population rescaling, but conducting a complete factorial experimental design to identify the global optimum across all parameter combinations was not the focus. Developing an algorithm to optimize time steps and population scaling for parameter fitting is a worthwhile future project. Finally, our convergence results rely on the law of large numbers. In scenarios with extremely small populations (< 10 individuals), stochastic deviations in the Petri nets are a feature, not a bug, and direct comparison with ODEs becomes less meaningful.

## Figures and Tables

**FIGURE 11.**
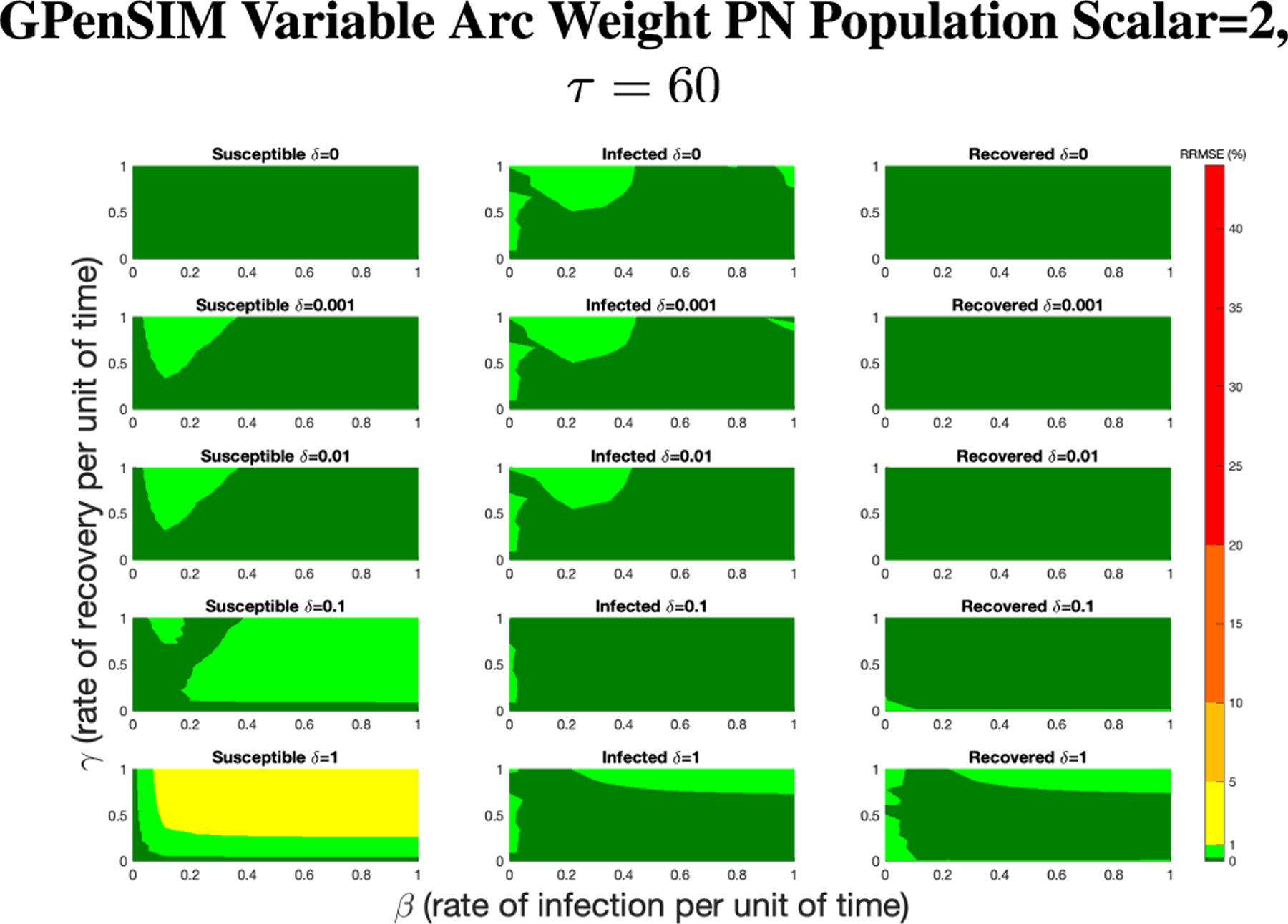
RRMSE percentage across parameter ranges of [0, 1] for each respective parameter with γ the y-axis of each subfigure, β the x-axis of each subfigure, and δ set at a different fixed value for each subfigure. PN Time Step Per Unit of Time parameter τ=60. Note that yellow is RRMSE ≤ 5%, light green is RRMSE ≤ 1%, and dark green is RRMSE ≤ 0.1%.

**FIGURE 13.**
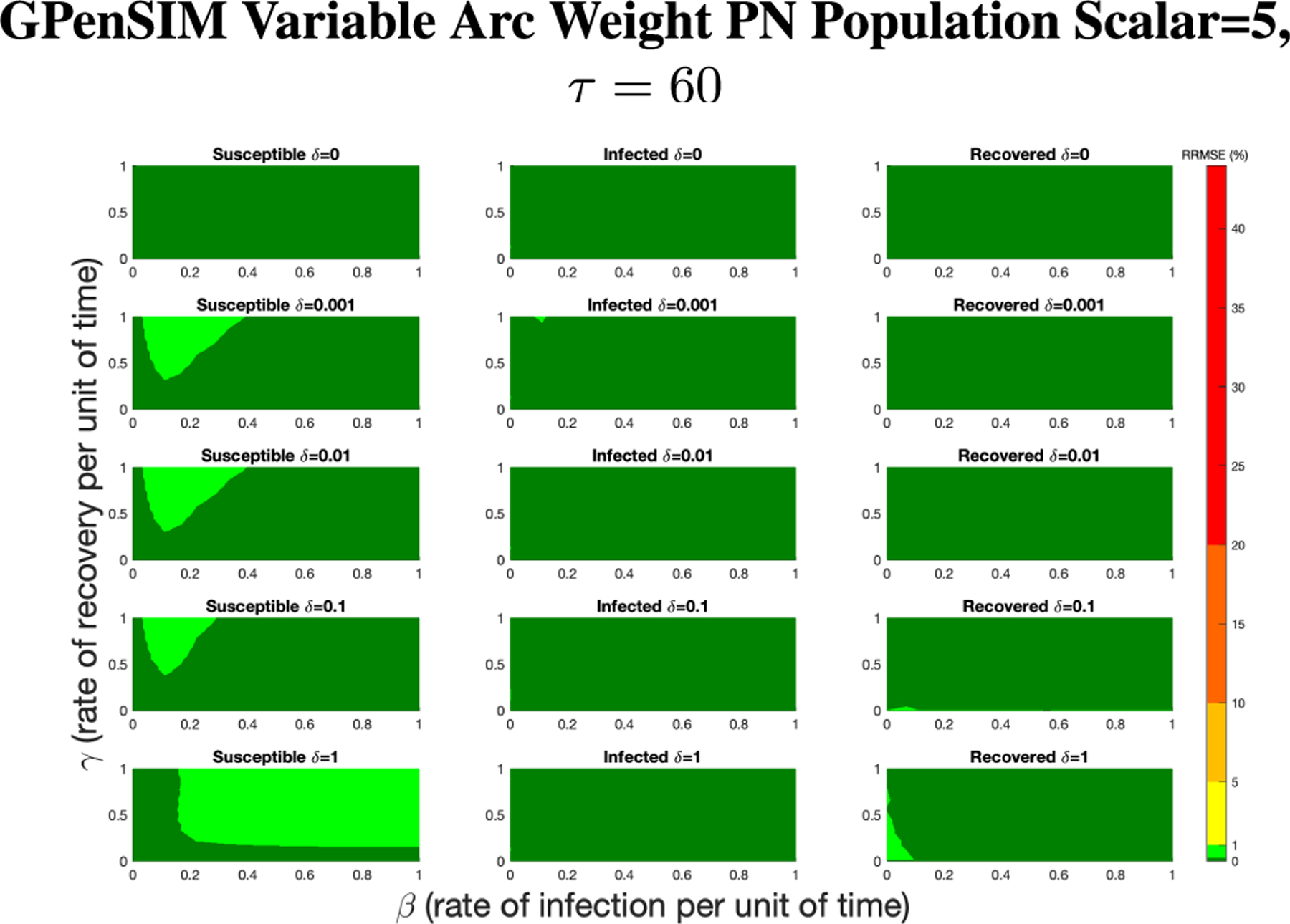
RRMSE percentage across parameter ranges of [0, 1] for each respective parameter with γ the y-axis of each subfigure, β the x-axis of each subfigure, and δ set at a different fixed value for each subfigure. PN Time Step Per Unit of Time parameter τ=60. Note that light green is RRMSE ≤ 1%, and dark green is RRMSE ≤ 0.1%.

**FIGURE 21.**
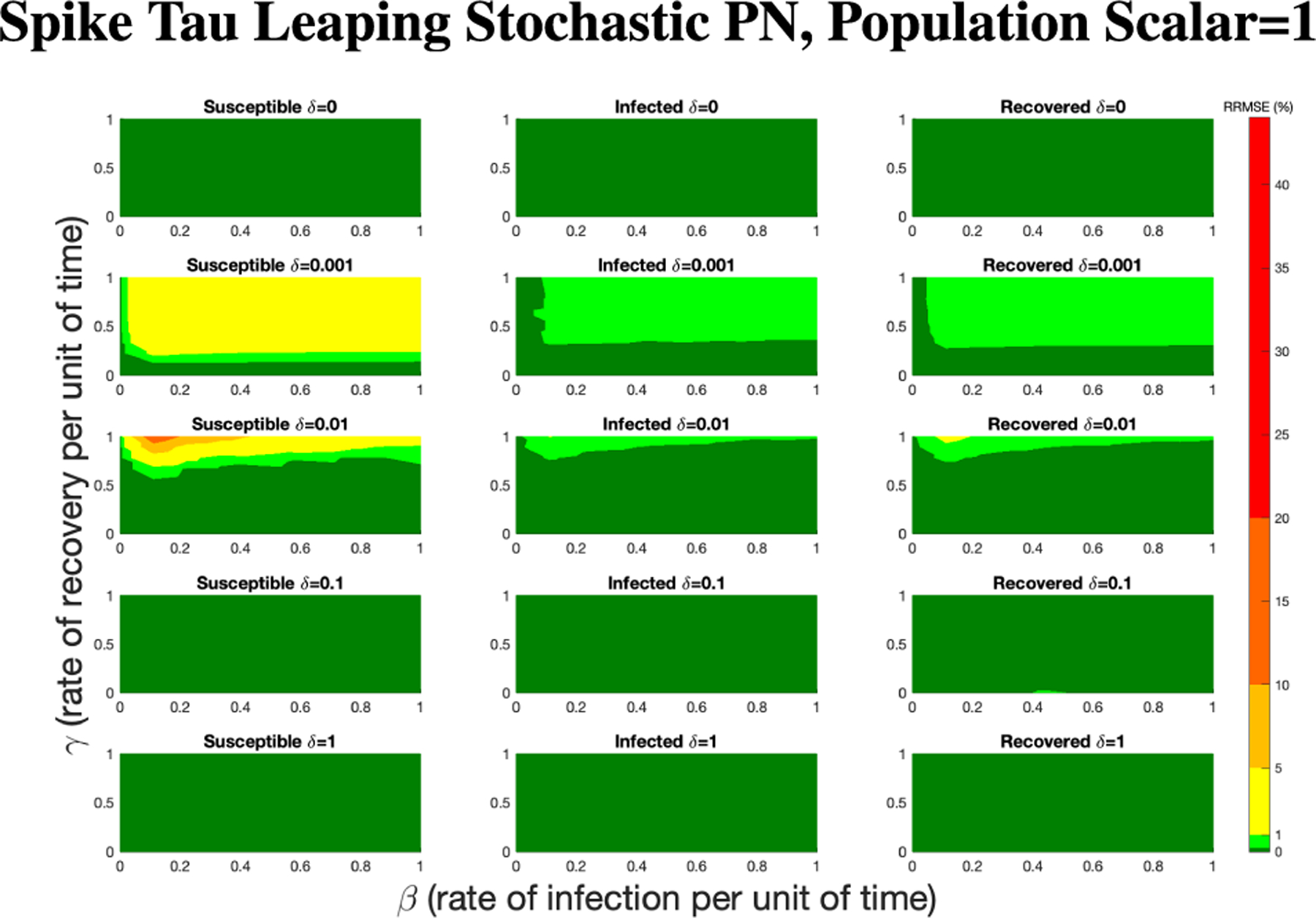
RRMSE percentage across parameter ranges of [0, 1] for each respective parameter with γ the y-axis of each subfigure, β the x-axis of each subfigure, and δ set at a different fixed value for each subfigure. Note that red is RRMSE ≤ 44% (43.75% being the max observed RRMSE across all simulations), dark orange is RRMSE ≤ 20%, light orange is RRMSE ≤ 10%, yellow is RRMSE ≤ 5%, light green is RRMSE ≤ 1%, and dark green is RRMSE ≤ .1%.

**FIGURE 22.**
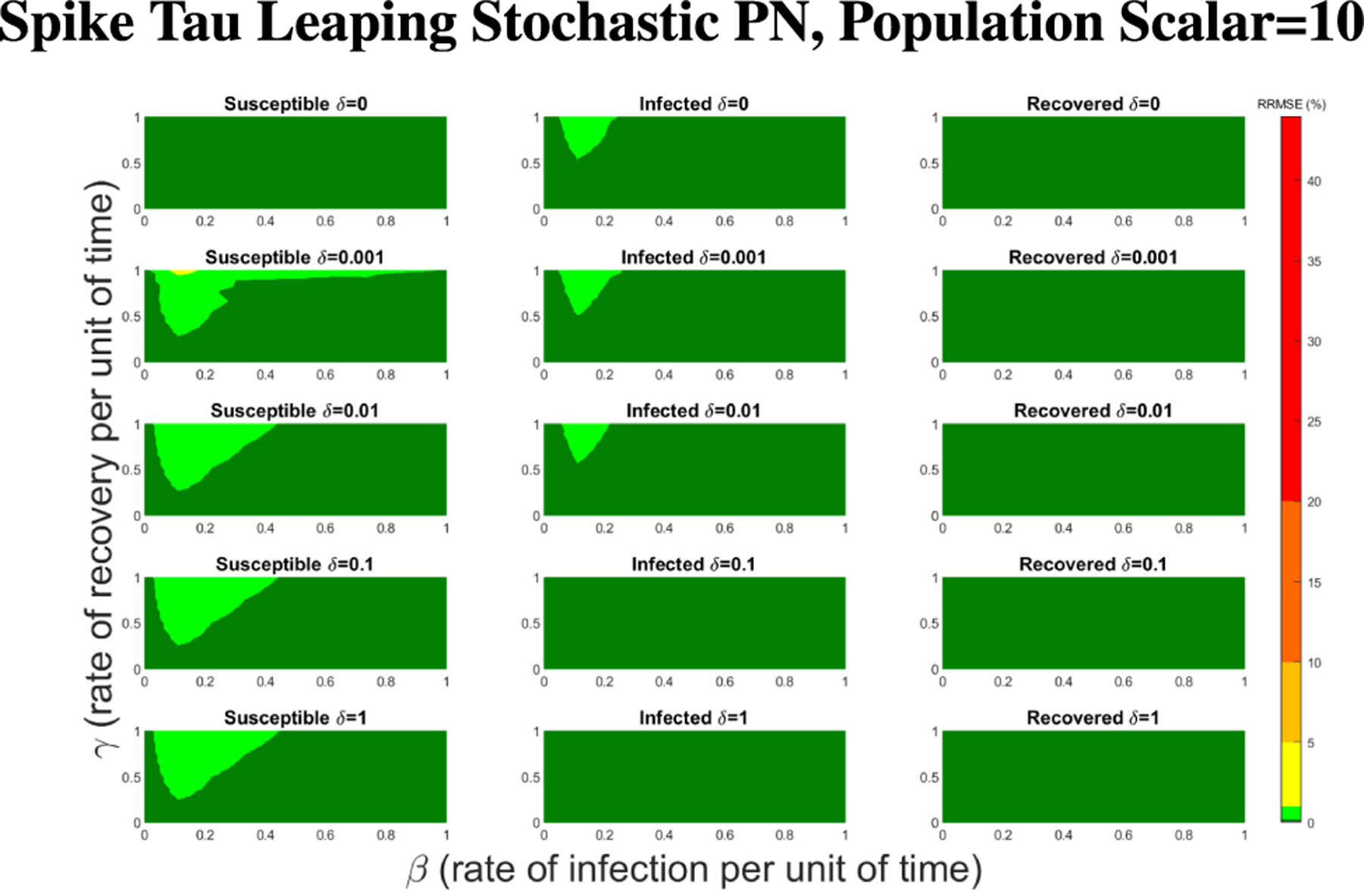
RRMSE percentage across parameter ranges of [0, 1] for each respective parameter with γ the y-axis of each subfigure, β the x-axis of each subfigure, and δ set at a different fixed value for each subfigure. Note that yellow is RRMSE ≤ 5%, light green is RRMSE ≤ 1%, and dark green is RRMSE ≤ .1%.

**FIGURE 23.**
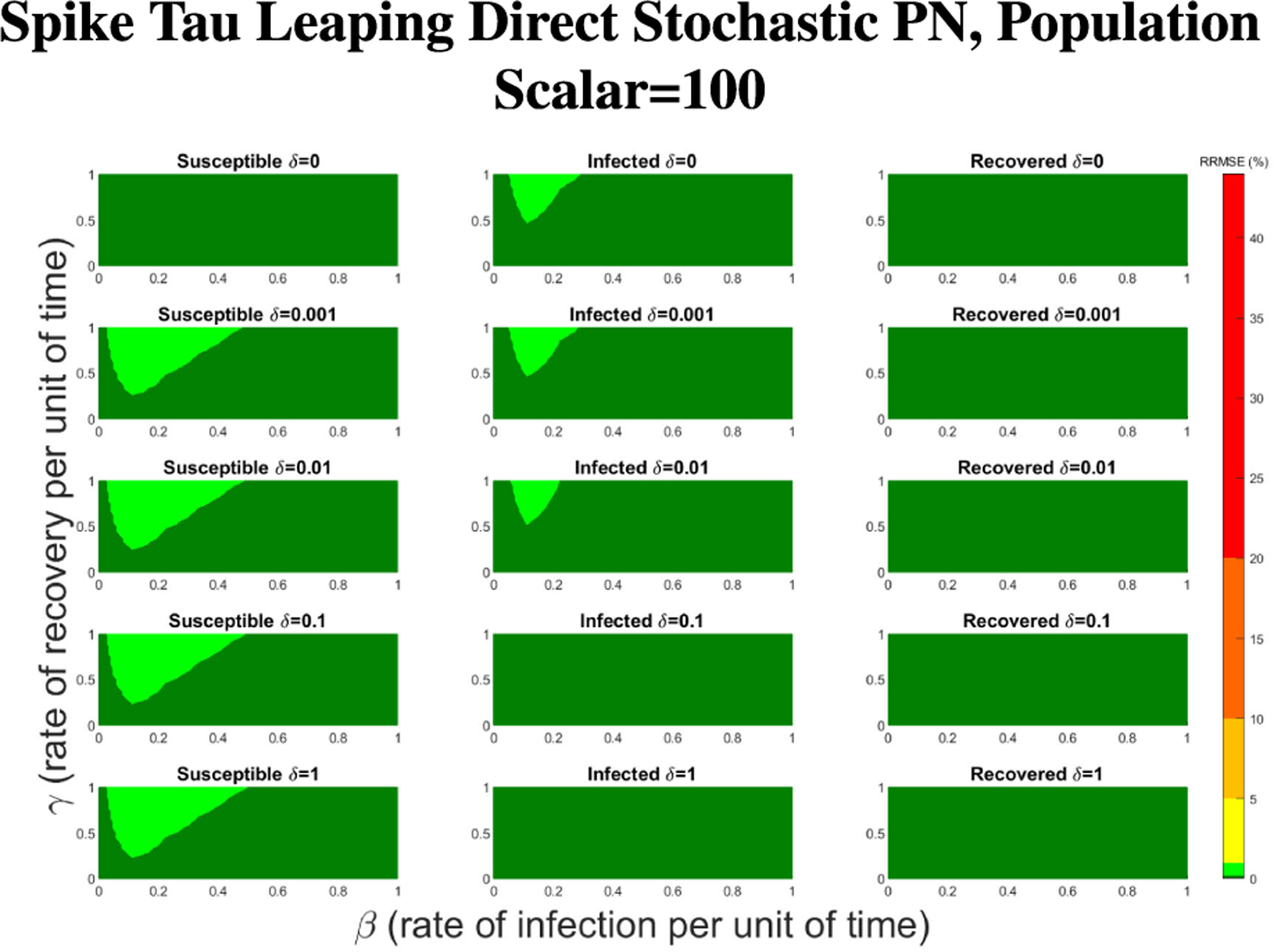
RRMSE percentage across parameter ranges of [0, 1] for each respective parameter with γ the y-axis of each subfigure, β the x-axis of each subfigure, and δ set at a different fixed value for each subfigure. Note that light green is RRMSE ≤ 1%, and dark green is RRMSE ≤ .1%.

**FIGURE 28.**
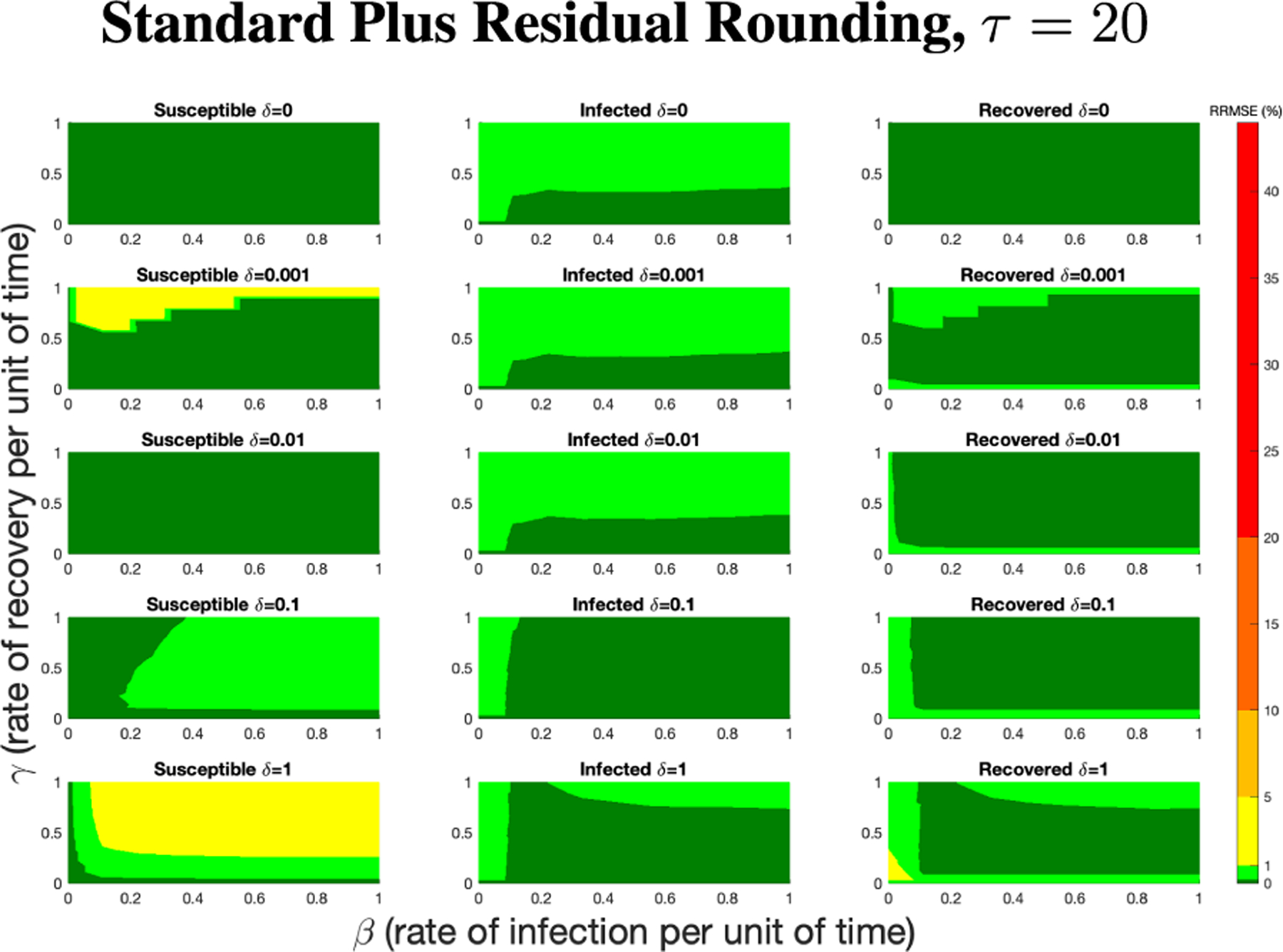
RRMSE percentage across parameter ranges of [0, 1] for each respective parameter with γ the y-axis of each subfigure, β the x-axis of each subfigure, and δ set at a different fixed value for each subfigure. PN Time Step Per Unit of Time parameter τ=1. Note that red is RRMSE ≤ 44% (43.75% being the max observed RRMSE across all simulations), dark orange is RRMSE ≤ 20%, light orange is RRMSE ≤ 10%, yellow is RRMSE ≤ 5%, light green is RRMSE ≤ 1%, and dark green is RRMSE ≤ .1%.

**FIGURE 29.**
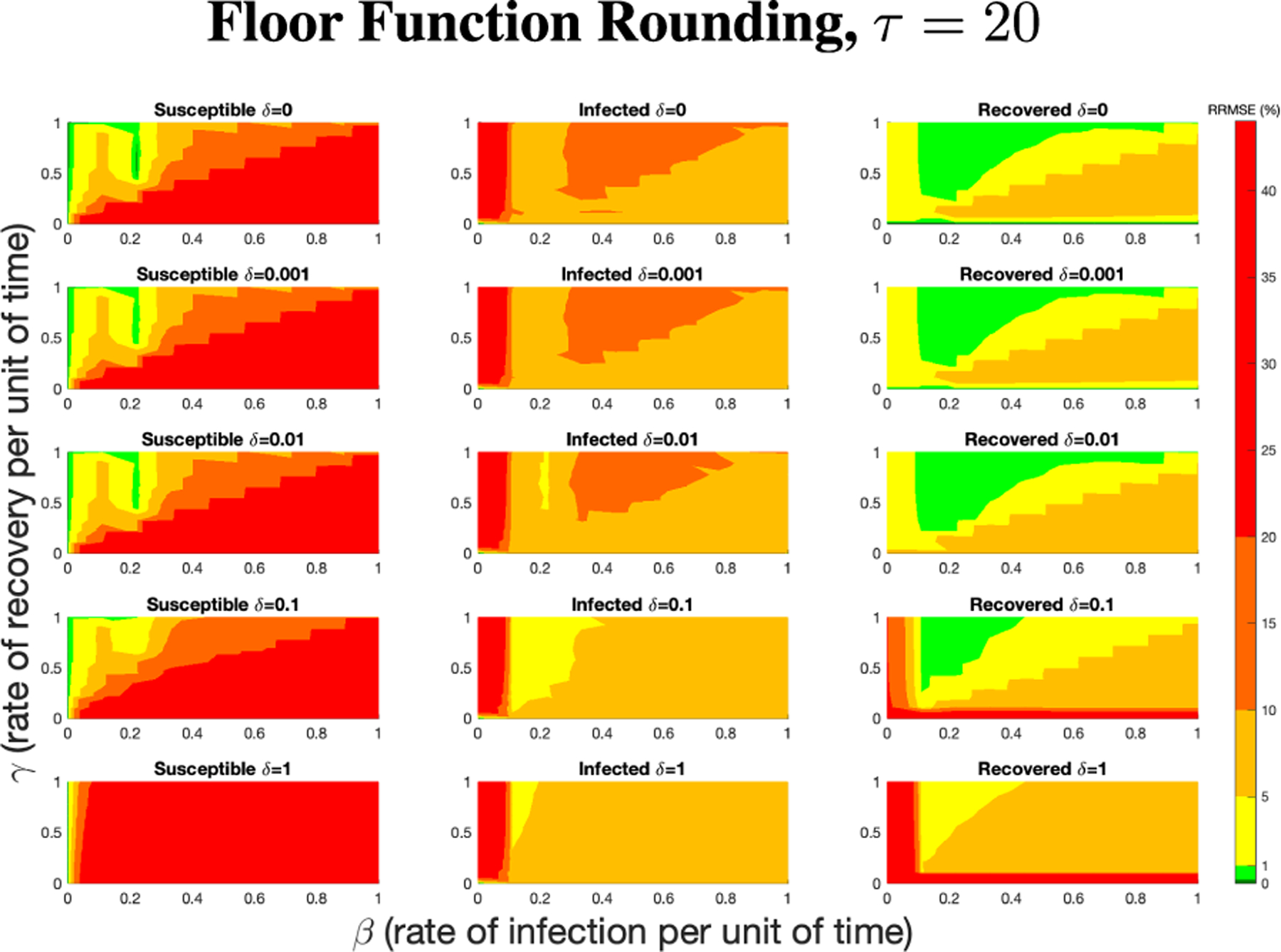
RRMSE percentage across parameter ranges of [0, 1] for each respective parameter with γ the y-axis of each subfigure, β the x-axis of each subfigure, and δ set at a different fixed value for each subfigure. PN Time Step Per Unit of Time parameter τ=1. Note that red is RRMSE ≤ 44% (43.75% being the max observed RRMSE across all simulations), dark orange is RRMSE ≤ 20%, light orange is RRMSE ≤ 10%, yellow is RRMSE ≤ 5%, light green is RRMSE ≤ 1%, and dark green is RRMSE ≤ .1%.

**FIGURE 30.**
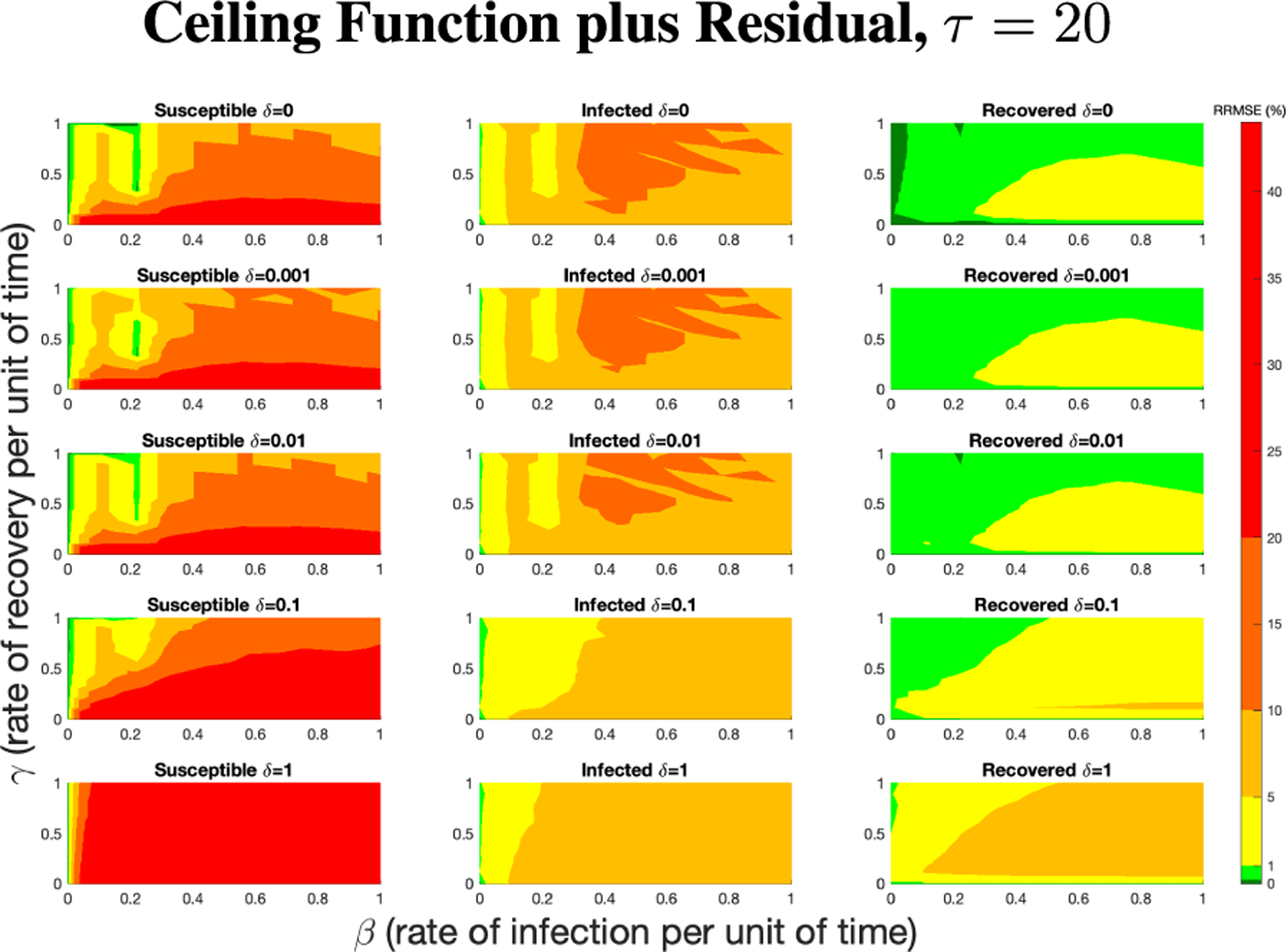
RRMSE percentage across parameter ranges of [0, 1] for each respective parameter with γ the y-axis of each subfigure, β the x-axis of each subfigure, and δ set at a different fixed value for each subfigure. PN Time Step Per Unit of Time parameter τ=1. Note that red is RRMSE ≤ 44% (43.75% being the max observed RRMSE across all simulations), dark orange is RRMSE ≤ 20%, light orange is RRMSE ≤ 10%, yellow is RRMSE ≤ 5%, light green is RRMSE ≤ 1%, and dark green is RRMSE ≤ .1%.

**FIGURE 31.**
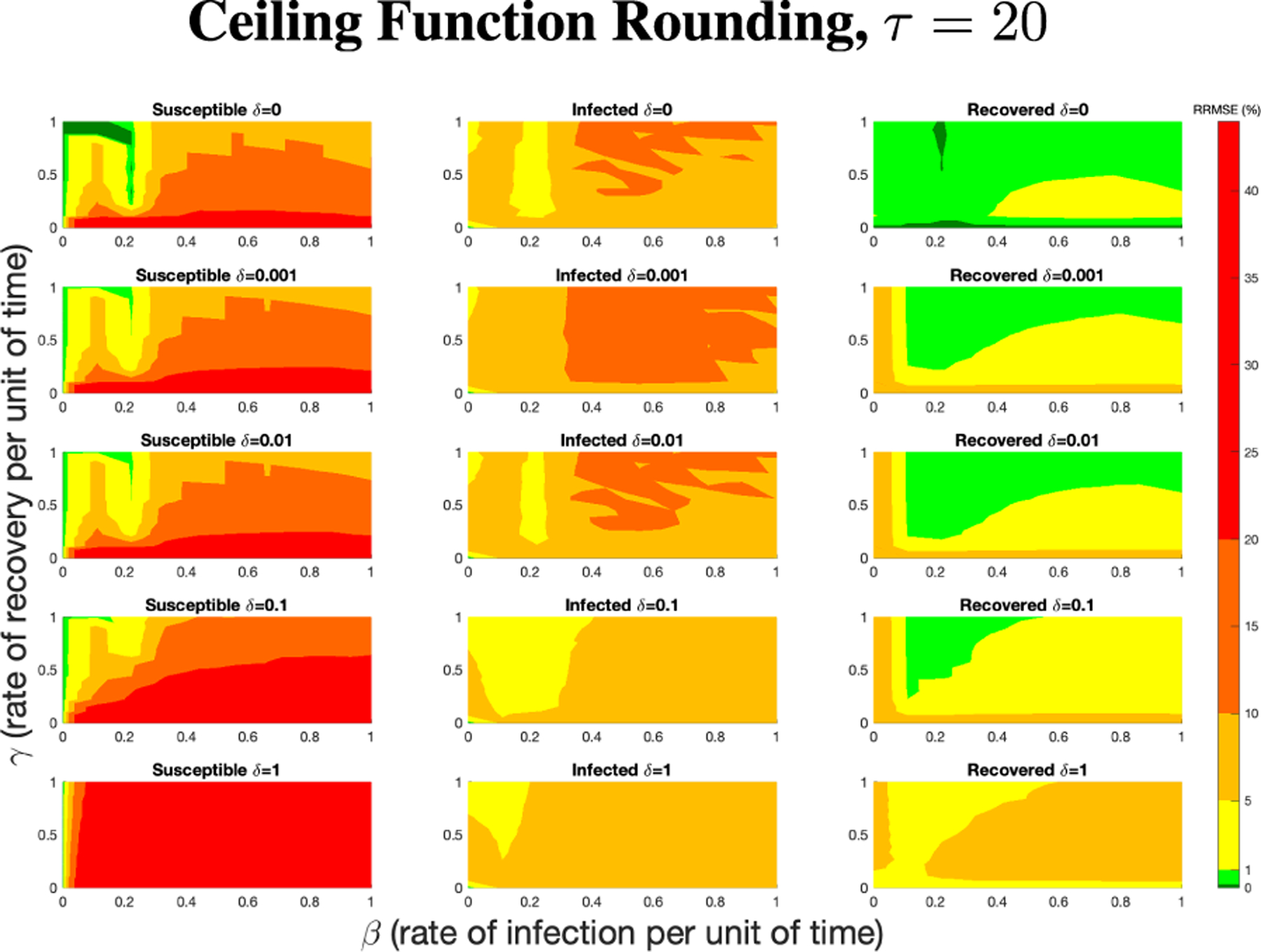
RRMSE percentage across parameter ranges of [0, 1] for each respective parameter with γ the y-axis of each subfigure, β the x-axis of each subfigure, and δ set at a different fixed value for each subfigure. PN Time Step Per Unit of Time parameter τ=1. Note that red is RRMSE ≤ 44% (43.75% being the max observed RRMSE across all simulations), dark orange is RRMSE ≤ 20%, light orange is RRMSE ≤ 10%, yellow is RRMSE ≤ 5%, light green is RRMSE ≤ 1%, and dark green is RRMSE ≤ .1%.

**FIGURE 32.**
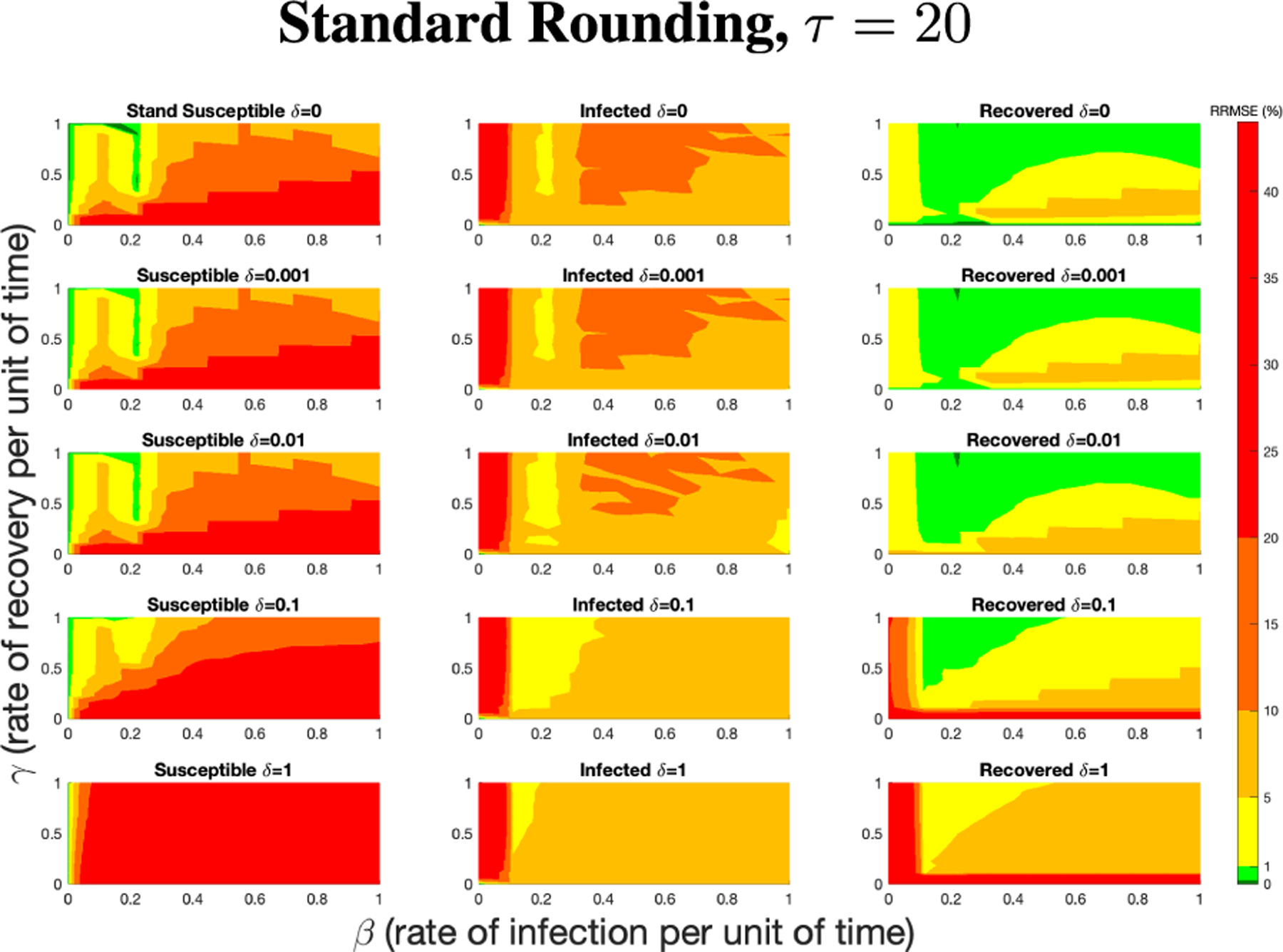
RRMSE percentage across parameter ranges of [0, 1] for each respective parameter with γ the y-axis of each subfigure, β the x-axis of each subfigure, and δ set at a different fixed value for each subfigure. PN Time Step Per Unit of Time parameter τ=1. Note that red is RRMSE ≤ 44% (43.75% being the max observed RRMSE across all simulations), dark orange is RRMSE ≤ 20%, light orange is RRMSE ≤ 10%, yellow is RRMSE ≤ 5%, light green is RRMSE ≤ 1%, and dark green is RRMSE ≤ .1%.

**FIGURE 33.**
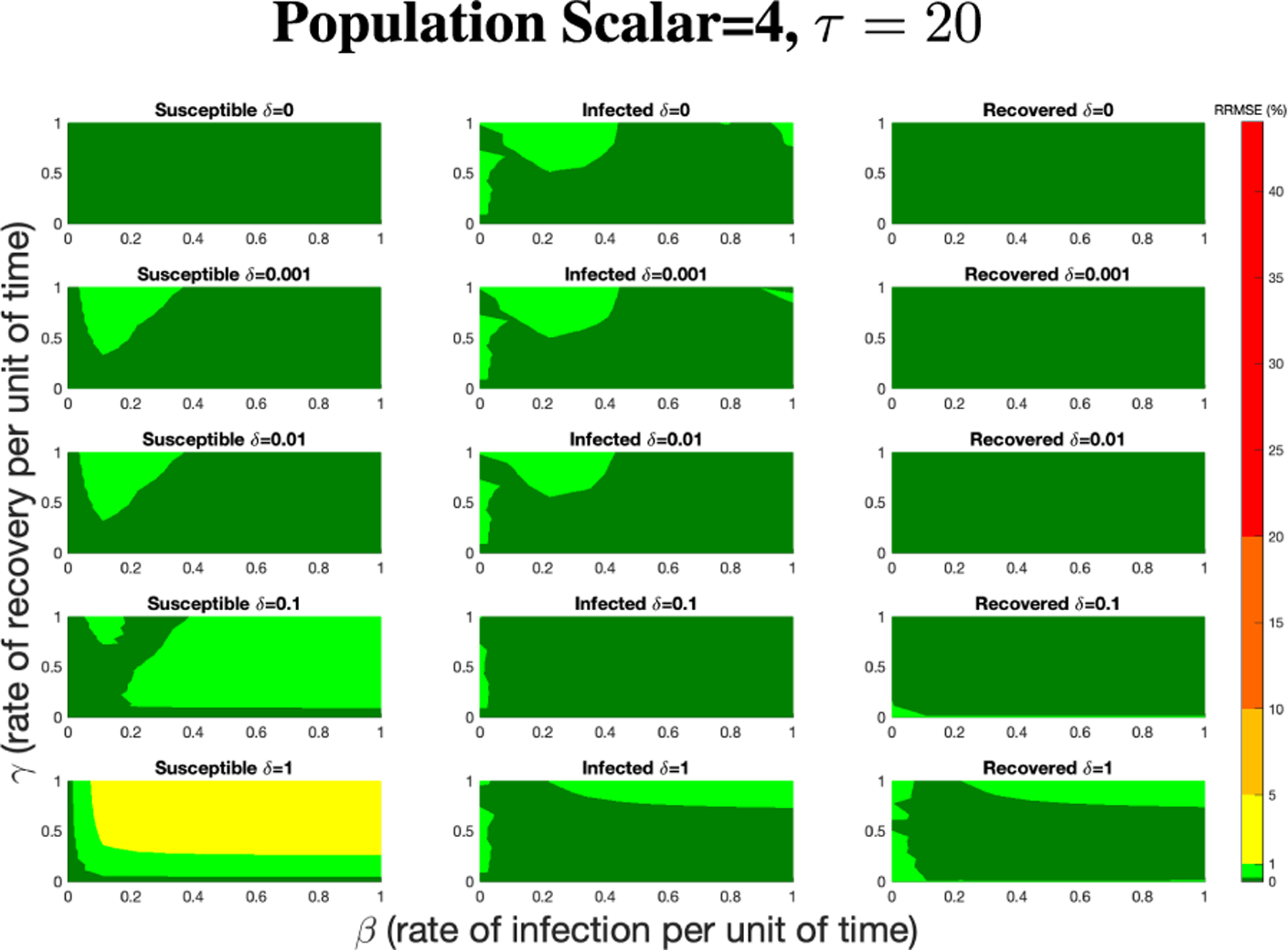
RRMSE percentage across parameter ranges of [0, 1] for each respective parameter with γ the y-axis of each subfigure, β the x-axis of each subfigure, and δ set at a different fixed value for each subfigure. PN Time Step Per Unit of Time parameter τ=1. Note that red is RRMSE ≤ 44% (43.75% being the max observed RRMSE across all simulations), dark orange is RRMSE ≤ 20%, light orange is RRMSE ≤ 10%, yellow is RRMSE ≤ 5%, light green is RRMSE ≤ 1%, and dark green is RRMSE ≤ .1%.

**FIGURE 34.**
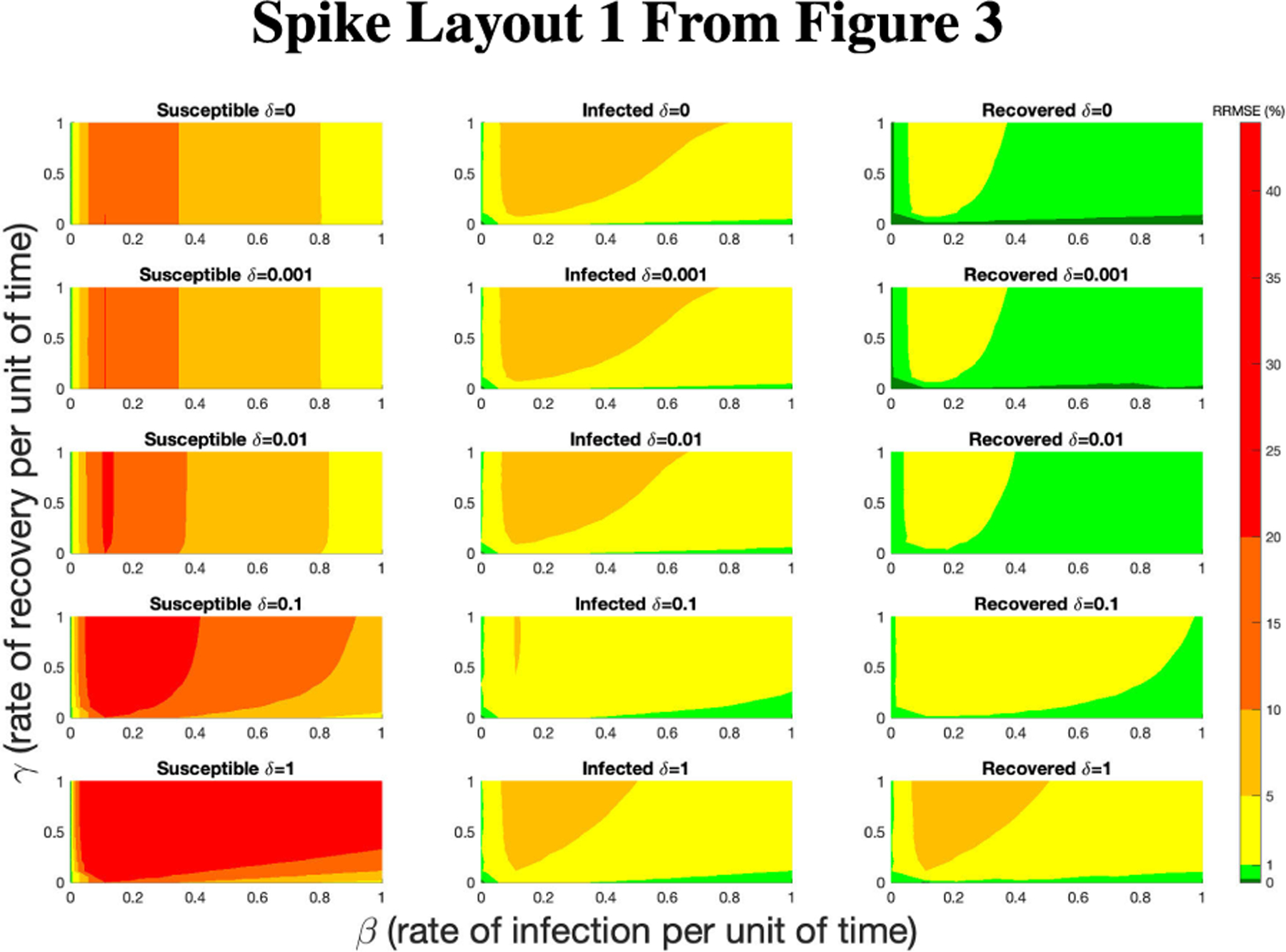
RRMSE percentage across parameter ranges of [0, 1] for each respective parameter with γ the y-axis of each subfigure, β the x-axis of each subfigure, and δ set at a different fixed value for each subfigure. PN Time Step Per Unit of Time parameter τ=1. Note that red is RRMSE ≤ 44% (43.75% being the max observed RRMSE across all simulations), dark orange is RRMSE ≤ 20%, light orange is RRMSE ≤ 10%, yellow is RRMSE ≤ 5%, light green is RRMSE ≤ 1%, and dark green is RRMSE ≤ .1%.

**FIGURE 35.**
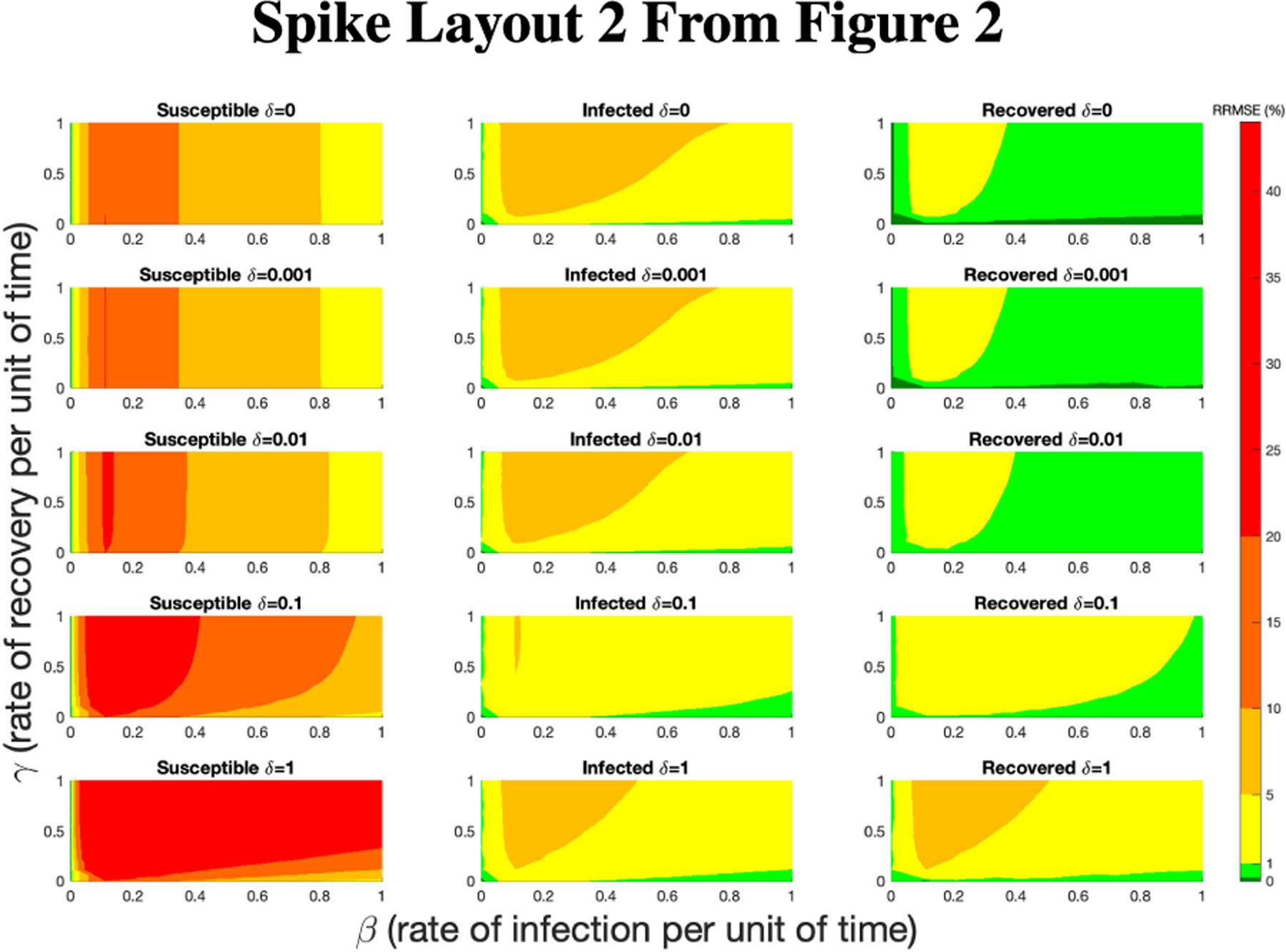
RRMSE percentage across parameter ranges of [0, 1] for each respective parameter with γ the y-axis of each subfigure, β the x-axis of each subfigure, and δ set at a different fixed value for each subfigure. PN Time Step Per Unit of Time parameter τ=1. Note that red is RRMSE ≤ 44% (43.75% being the max observed RRMSE across all simulations), dark orange is RRMSE ≤ 20%, light orange is RRMSE ≤ 10%, yellow is RRMSE ≤ 5%, light green is RRMSE ≤ 1%, and dark green is RRMSE ≤ .1%.

**FIGURE 36.**
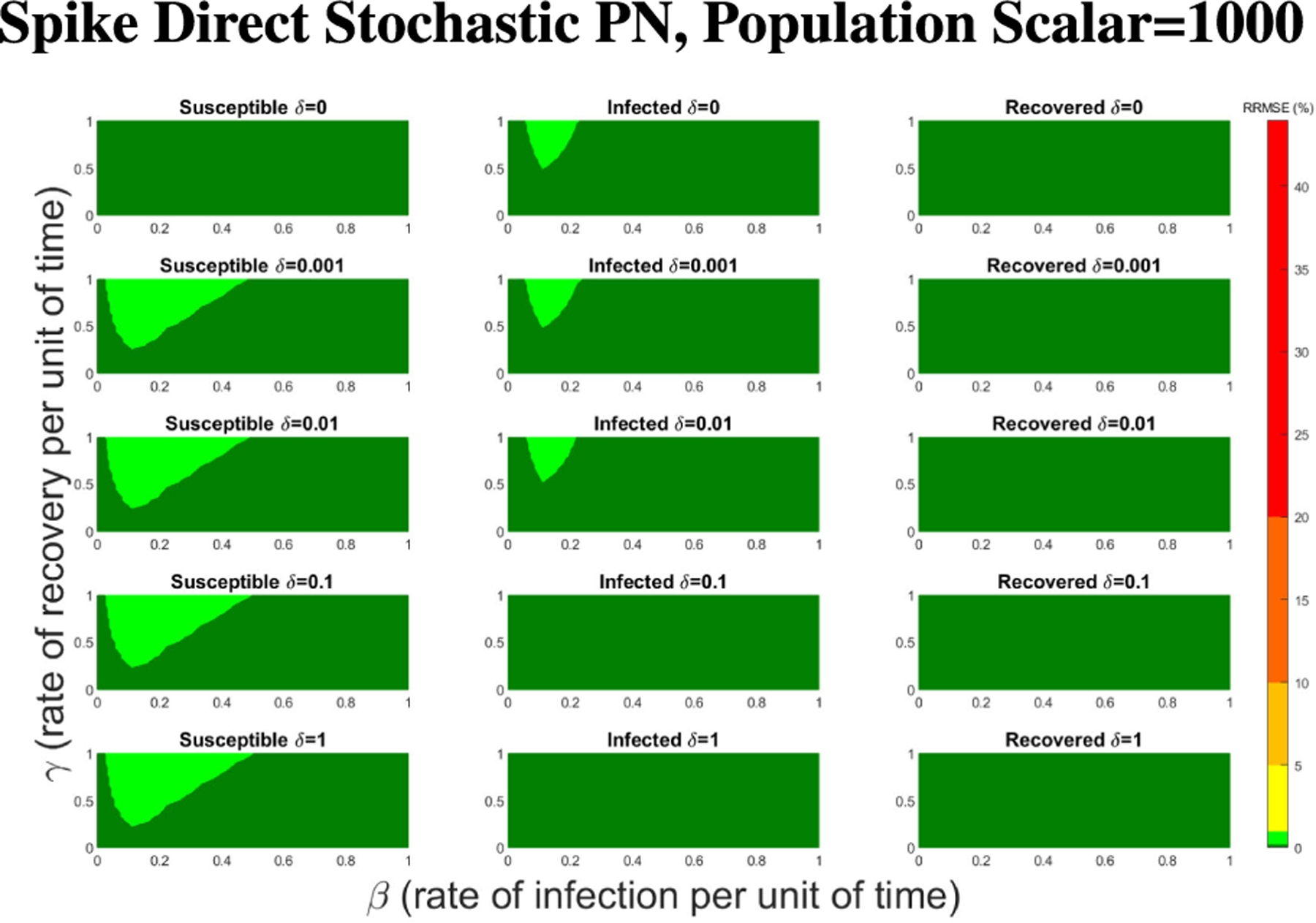
RRMSE percentage across parameter ranges of [0, 1] for each respective parameter with γ the y-axis of each subfigure, β the x-axis of each subfigure, and δ set at a different fixed value for each subfigure. Note that light orange is RRMSE ≤ 10%, yellow is RRMSE ≤ 5%, light green is RRMSE ≤ 1%, and dark green is RRMSE ≤ .1%.

**FIGURE 37.**
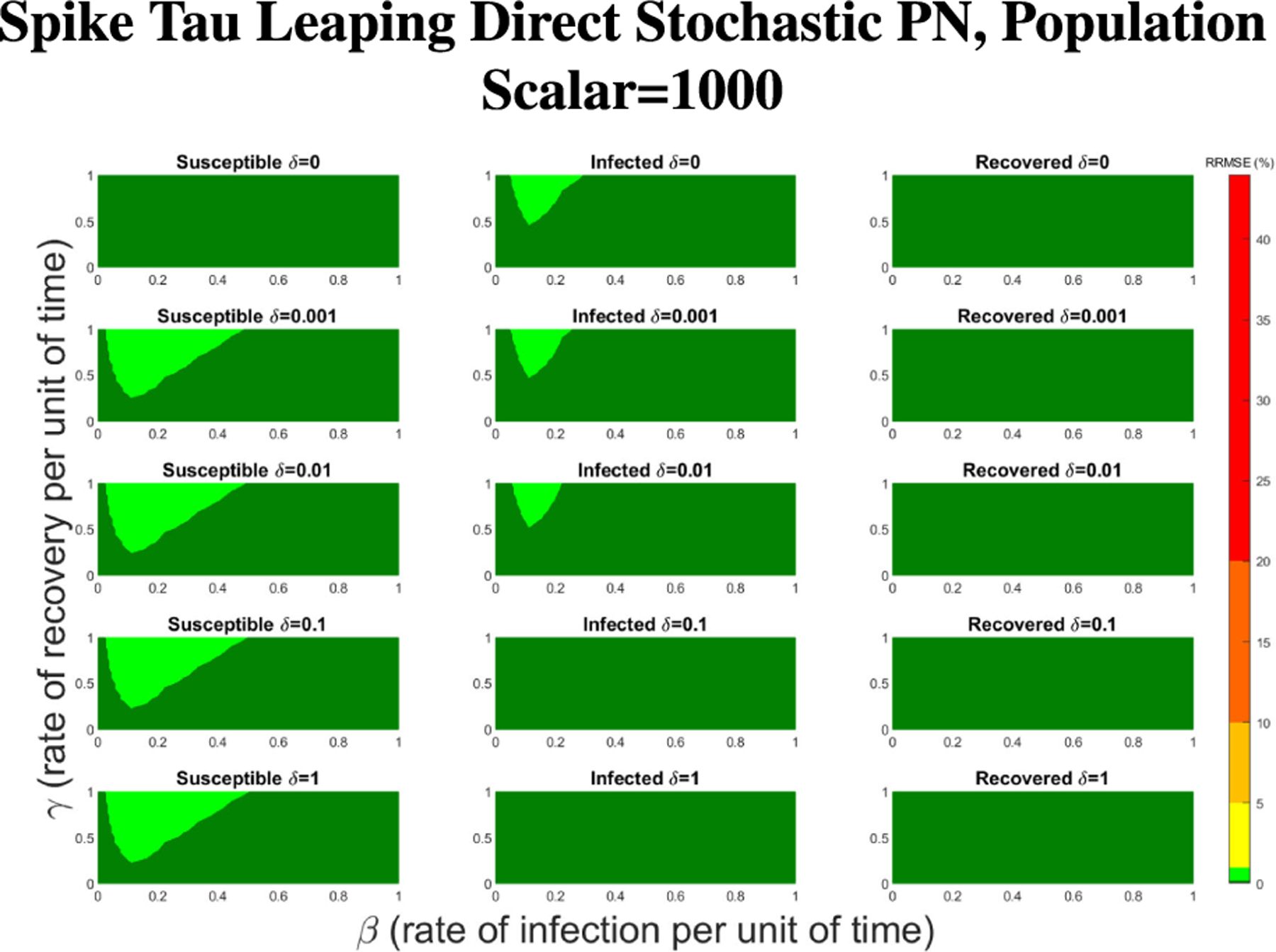
RRMSE percentage across parameter ranges of [0, 1] for each respective parameter with γ the y-axis of each subfigure, β the x-axis of each subfigure, and δ set at a different fixed value for each subfigure. Note that light orange is RRMSE ≤ 10%, yellow is RRMSE ≤ 5%, light green is RRMSE ≤ 1%, and dark green is RRMSE ≤ .1%.

**TABLE 1.** This table provides reference for ODE and PN parameters and initial conditions. These initial populations levels can be scaled as explained in Section III-C.

Parameter	Description	Reference Range/Value
S(0)	Initial Susceptible	1000
I(0)	Initial Infected	10
R(0)	Initial Recovered	10
β	Infection Rate	[0, 1]
γ	Recovery Rate	[0, 1]
δ	Re-susceptibility Rate	[0, 1]
N	Total Population	1020

## V. SUPPLEMENTARY INDEX

The Supplementary or Appendix section (Figure 26–37) shows additional plots from our simulations including τ runs in GPENSIM, rounding methods in GPENSIM, pop scalar/τ runs in GPENSIM, spike simulations for Layout 1 and 2 and spike simulations with population scalars.

### A. ADDITIONAL τ RUNS IN GPENSIM

Figures 26 and 27 are additional runs of GPenSim with different τ values of 40 and 80 respectively (population scalar remains 1).

### B. ROUNDING METHOD RUNS GPENSIM

Figures 28–32 show different rounding method RRMSE at τ=20 and a population scalar of 1. These figures helped in determining the best rounding method.

### C. SPIKE STOCHASTIC PETRI NETS SIMULATIONS

Figures 34 and 35 show RRMSE for Delta leaping method within Spike. Delta leaping is a stochastic PN method option in Spike we determined to not be suitable based on the following figures.

### D. SPIKE STOCHASTIC PETRI NETS SIMULATIONS WITH POPULATION SCALARS

Figures 36 and 37 show Direct and Tau leaping method within Spike with population scalar of 1000.
